# Pathogenic *ARH3* mutations result in ADP-ribose chromatin scars during DNA strand break repair

**DOI:** 10.1038/s41467-020-17069-9

**Published:** 2020-07-07

**Authors:** Hana Hanzlikova, Evgeniia Prokhorova, Katerina Krejcikova, Zuzana Cihlarova, Ilona Kalasova, Jan Kubovciak, Jana Sachova, Richard Hailstone, Jan Brazina, Shereen Ghosh, Sebahattin Cirak, Joseph G. Gleeson, Ivan Ahel, Keith W. Caldecott

**Affiliations:** 1 Department of Genome Dynamics, Institute of Molecular Genetics of the Czech Academy of Sciences, Prague 4, 142 20 Czech Republic; 2 Genome Damage and Stability Centre, School of Life Sciences, University of Sussex, Falmer, Brighton, BN1 9RQ UK; 3 Sir William Dunn School of Pathology, University of Oxford, Oxford, OX1 3RE UK; 4 Department of Genomics and Bioinformatics, Institute of Molecular Genetics of the Czech Academy of Sciences, Prague 4, 142 20 Czech Republic; 5 Laboratory for Pediatric Brain Disease, Howard Hughes Medical Institute, University of California, San Diego, La Jolla, CA 92093 USA; 6 Rady Children’s Institute for Genomic Medicine, Rady Children’s Hospital, San Diego, CA 92123 USA; 7 Center for Molecular Medicine Cologne, University of Cologne, Cologne, 50931 Germany; 8 Department of Pediatrics, Faculty of Medicine and University Hospital Cologne, University of Cologne, Cologne, 50931 Germany; 9 Center for Rare Diseases, Faculty of Medicine and University Hospital Cologne, University of Cologne, Cologne, 50931 Germany

**Keywords:** Mechanisms of disease, DNA damage and repair

## Abstract

Neurodegeneration is a common hallmark of individuals with hereditary defects in DNA single-strand break repair; a process regulated by poly(ADP-ribose) metabolism. Recently, mutations in the ARH3 *(ADPRHL2)* hydrolase that removes ADP-ribose from proteins have been associated with neurodegenerative disease. Here, we show that *ARH3*-mutated patient cells accumulate mono(ADP-ribose) scars on core histones that are a molecular memory of recently repaired DNA single-strand breaks. We demonstrate that the ADP-ribose chromatin scars result in reduced endogenous levels of important chromatin modifications such as H3K9 acetylation, and that ARH3 patient cells exhibit measurable levels of deregulated transcription. Moreover, we show that the mono(ADP-ribose) scars are lost from the chromatin of ARH3-defective cells in the prolonged presence of PARP inhibition, and concomitantly that chromatin acetylation is restored to normal. Collectively, these data indicate that ARH3 can act as an eraser of ADP-ribose chromatin scars at sites of PARP activity during DNA single-strand break repair.

## Abstract (web summary)

Defects in DNA single-strand break repair are associated with neurodegenerative disease. Here the authors reveal that mutations in *ARH3* interfere with the catabolism of mono-(ADP-ribose) and lead to its accumulation on core histones following repair of endogenous or exogenous DNA single-strand breaks.

## Introduction

ADP-ribosylation is a ubiquitous post-translational modification that regulates numerous processes including the repair of DNA strand breaks arising stochastically or during normal DNA replication^1–3^. Cells possess three poly(ADP-ribose) polymerase (PARP) enzymes (namely, PARP1, PARP2, and PARP3) that detect DNA strand breaks and signal their presence by catalysing the rapid autoribosylation and transribosylation of proteins, including histones, with mono(ADP-ribose) and/or poly(ADP-ribose). Of the three single-strand break (SSB)-inducible PARPs identified to date PARP1 is the founding member and the best characterised, and accounts for more than 80% of total PARP activity in cells. Despite the abundance of PARP1 most of the poly(ADP-ribose) generated at SSBs is rapidly degraded with a half-life of several minutes or less, by poly(ADP-ribose) glycohydrolase (PARG)^4,5^. Although PARG can efficiently cleave the ribose–ribose links that constitute poly(ADP-ribose) chains, it cannot remove the terminal (protein-proximal) ADP-ribose moiety, and thereby generates mono(ADP-ribosylated) proteins. As a result, the two mono(ADP-ribose) glycohydrolases ARH3 and TARG are required to remove protein-proximal ADP-ribose moieties from ribosylated serine and glutamate/aspartate residues in histones and other proteins, respectively^6–9^.

The role of poly(ADP-ribosylation) at sites of SSBs includes the modulation of chromatin structure and promoting the recruitment of XRCC1 protein complexes that accelerate DNA single-strand break repair (SSBR)^2,10,11^. Hereditary mutations in components of SSBR are associated with neurodevelopmental and neurodegenerative pathologies, including progressive cerebellar ataxia and, in severe cases, seizures^12,13^. Recently, mutations in XRCC1, the scaffold protein that interacts with enzymatic components of each of the core steps of SSBR, were associated with progressive cerebellar ataxia^14,15^. Intriguingly, the neurological consequences of conditional *Xrcc1* deletion in mouse, which include cerebellar ataxia, seizures and shortened life-span, are in part the result of Parp1 hyperactivation^15,16^, highlighting the likely pathological consequences of aberrant and/or excessive PARP signalling at unrepaired SSBs.

Similar to defects in SSBR, aberrant ADP-ribose metabolism is also linked to neurodegeneration in humans. This is illustrated by the genetic disease childhood-onset, stress-induced, with variable ataxia and seizures (CONDSIAS), which is mutated in the *ADPRHL2* gene encoding ARH3^17–19^. That ARH3 mutations might trigger neurodegeneration by perturbing ADP-ribose metabolism during SSBR is consistent with reported involvement of this protein in degrading free poly(ADP-ribose) chains produced following H_2_O_2_-induced oxidative stress, a major inducer of SSBs, and by the protection against oxidative stress afforded in *ARH3*^*−/−*^ cells and mice by PARP inhibition^19,20^. In addition, sustained depletion of PARG reduces the rate of SSBR^21^ raising the possibility that ARH3 mutation or deletion might similarly slow SSBR, directly.

Here, we address the impact of ARH3 mutation or deletion on SSBR and chromatin ADP-ribosylation. We find that while the absence of ARH3 hydrolase does not impede SSBR, it leads to the persistence of mono(ADP-ribose) chromatin scars at sites of SSBs that have long since been repaired. We suggest that these mono-ADP-ribose scars accumulate and impede local histone acetylation and other histone modifications in ARH3-mutated cells, resulting in a perturbed histone code, deregulated transcription, and cellular dysfunction.

## Results and discussion

### Normal rates of SSBR in *ARH3*-mutated patient fibroblasts

**Fig. 1:**
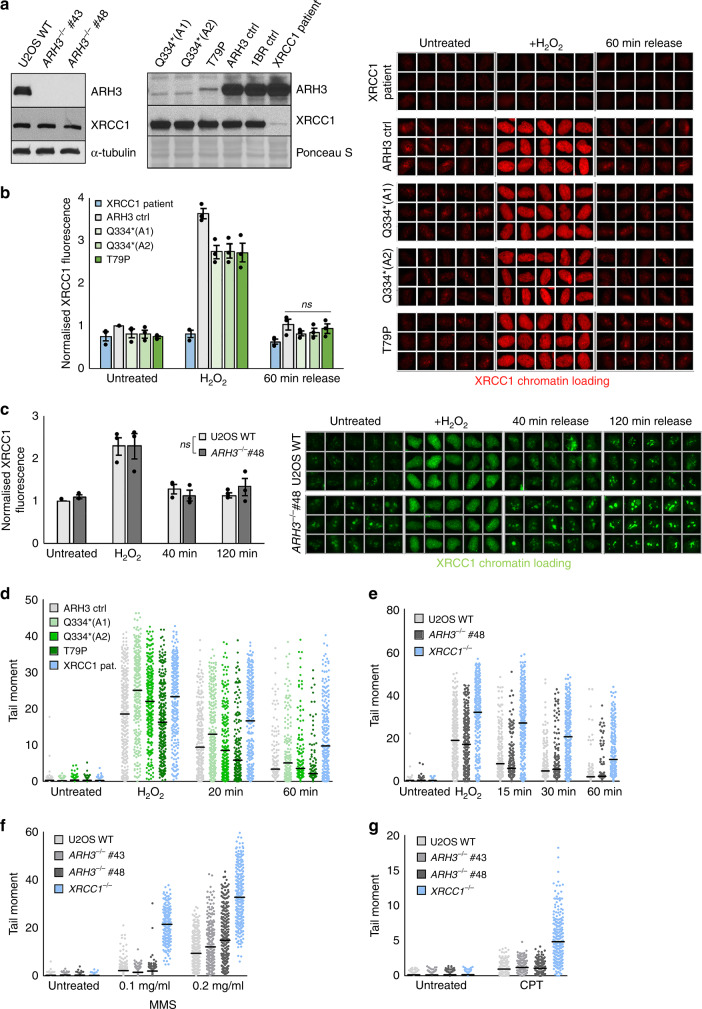
Normal rates of DNA single-strand break repair in ARH3-defective cells. **a** ARH3 and XRCC1 protein levels in the indicated U2OS (left) and patient-derived fibroblasts (right) were measured by western blotting. **b** XRCC1 chromatin binding measured by indirect immunofluorescence in detergent pre-extracted control and patient fibroblasts before, immediately after 10 min treatment with 150 μM H_2_O_2_ on ice, and after 60 min release in H_2_O_2_-free medium. Representative ScanR images (right) and quantification using ScanR software (left) are shown. Statistical analysis (two-tailed *t*-test) is indicated (ns not significant). **c** Similar experiment to (**b**); wild-type and *ARH3*^*−/−*^ U2OS cells were treated for 10 min or not with 2 mM H_2_O_2_ on ice, followed by a repair period of 40 min or 120 min in H_2_O_2_-free medium. Data are as in panel **b** and both are the mean ± SEM of three biologically independent experiments. Statistical analysis (two-way analysis of variance) is indicated. The samples are not significantly different (ns). **d** DNA strand breakage quantified by alkaline comet assays in the indicated control and patient fibroblasts before, immediately after treatment with 50 μM H_2_O_2_ on ice, and after the indicated repair periods in H_2_O_2_-free medium. **e** Similar experiment to **d**; wild-type, *ARH3*^*−/−*^, and *XRCC1*^*−/−*^ U2OS cells were treated with 100 μM H_2_O_2_ followed by a repair period of 15, 30, and 60 min in H_2_O_2_-free medium. **f** Alkaline comet tail moments in wild-type, *ARH3*^*−/−*^ and *XRCC1*^*−/−*^ U2OS cells before and after 20 min treatment with indicated doses of MMS. **g** Alkaline comet tail moments in wild-type, *ARH3*^*−/−*^ and *XRCC1*^*−/−*^ U2OS cells before and after 45 min treatment with 10 μM CPT. Data for **d**, **e**, **f**, and **g** are comet tail moments of 300 cells examined over three independent experiments (100 cells each), horizontal bars show the average. The only significantly different sample in each dataset is *XRCC1*^*−/−*^ U2OS (two-way analysis of variance). Representative pictures are shown in Supplementary Fig. 2.

Given the overlapping clinical phenotypes of *ARH3*-mutated and DNA single-strand break repair (SSBR) defective diseases we postulated that *ARH3* mutations might result in defects in this DNA repair process. This would be consistent with the established importance of poly(ADP-ribose) metabolism for SSBR^21,22^. To address this question, we employed primary human fibroblasts from three different *ARH3*-mutated patients (A1, A2, and T79P), harbouring the homozygous mutations Q334* (A1 and A2) or T79P, and two unaffected controls (ARH3 ctrl and 1BR)^15,17^. As an additional control, we also used U2OS cells in which *ARH3* was inactivated by CRISPR/Cas9 gene editing (clones #43 and #48)^7^. As expected, the *ARH3*^*−/−*^ U2OS cells lacked detectable levels of ARH3 protein (Fig. 1a, left). In contrast, all three patient fibroblasts appeared to possess a small amount of residual ARH3 protein, as detected by anti-ARH3 antibody (Fig. 1a, right). This protein was ARH3 because it was further reduced by ARH3 siRNA (Supplementary Fig. 1a). Moreover, the residual ARH3 in the fibroblasts from affected siblings A1 and A2 migrated slightly faster than wild-type ARH3, consistent with the Q334* mutation in this family, which results in a premature stop codon and loss of the C-terminal 30 amino acids^17^. In contrast to ARH3, other critical enzymes involved in ADP-ribose metabolism such as PARP1, PARP2, ARH1, and PARG were present at normal levels in ARH3 patient cells (Supplementary Fig. 1b).

Since one role of poly(ADP-ribose) during SSBR involves recruitment of the scaffold protein XRCC1^11,23,24^ we compared the level of this protein in the chromatin of control and ARH3 patient cells, and as a negative control in patient cells from an individual harbouring mutations in XRCC1^15^, following treatment with hydrogen peroxide (H_2_O_2_); a physiologically relevant source of SSBs induced by oxidative stress. The extent to which XRCC1 accumulated in oxidised chromatin during incubation with H_2_O_2_ was reduced slightly in ARH3 patient fibroblasts (Fig. 1b) when compared to the control fibroblasts from the unaffected father, but was unaffected in *ARH3*^*−/−*^ U2OS cells (Fig. 1c), as was the total level of XRCC1 (Fig. 1a and Supplementary Fig. 1c). More importantly, the level of XRCC1 in chromatin declined to background levels within 40–60 min in both control and ARH3-defective cells during a subsequent incubation in H_2_O_2_-free medium (Fig. 1b, c). Since the presence of XRCC1 in chromatin is a hallmark of SSBR, these results suggested that the rate of SSBR was largely unaffected by loss of ARH3 activity.

To address the relationship between ARH3 and SSBR more directly, we next measured the induction and repair of H_2_O_2_-induced DNA strand breaks using alkaline comet assays. Indeed, consistent with the above conclusion, the rate at which DNA breaks declined following treatment with H_2_O_2_ was similar in control and all three ARH3 patient fibroblasts (Fig. 1d and Supplementary Fig. 2a). This contrasted with XRCC1 patient cells in which the rate of DNA strand break repair was markedly reduced, as expected (Fig. 1d and Supplementary Fig. 2a). Similar results were observed if we compared wild-type, *ARH3*^*−/−*^ and *XRCC1*^*−/−*^ U2OS cells (Fig. 1e and Supplementary Fig. 2b). Collectively, these results indicate that ARH3 is dispensable for normal rates of SSBR in human cells, following oxidative stress.

To explore the possible role of ARH3 in SSBR further, we compared wild-type, *XRCC1*^*−/−*^ and *ARH3*^*−/−*^ U2OS cells for levels of SSBs following treatment with methyl methanesulfonate (MMS), an alkylating agent in which SSBs arise during DNA base excision repair (BER). Whereas *XRCC1*^*−/−*^ U2OS cells accumulated high levels of DNA strand breaks during BER, *ARH3*^*−/−*^ U2OS cells once again exhibited SSB levels that were similar to wild-type (Fig. 1f and Supplementary Fig. 2c). Similar results were observed following the treatment of these cells with camptothecin (CPT), indicating that ARH3 is also dispensable for the repair of SSBs induced by the abortive activity of DNA topoisomerase 1 (Fig. 1g and Supplementary Fig. 2d). Thus, we conclude from these experiments that ARH3 is dispensable for the repair of a broad range of physiologically relevant SSBs.

### Mono(ADP-ribosylation) in *ARH3*-mutated cells during SSBR

**Fig. 2:**
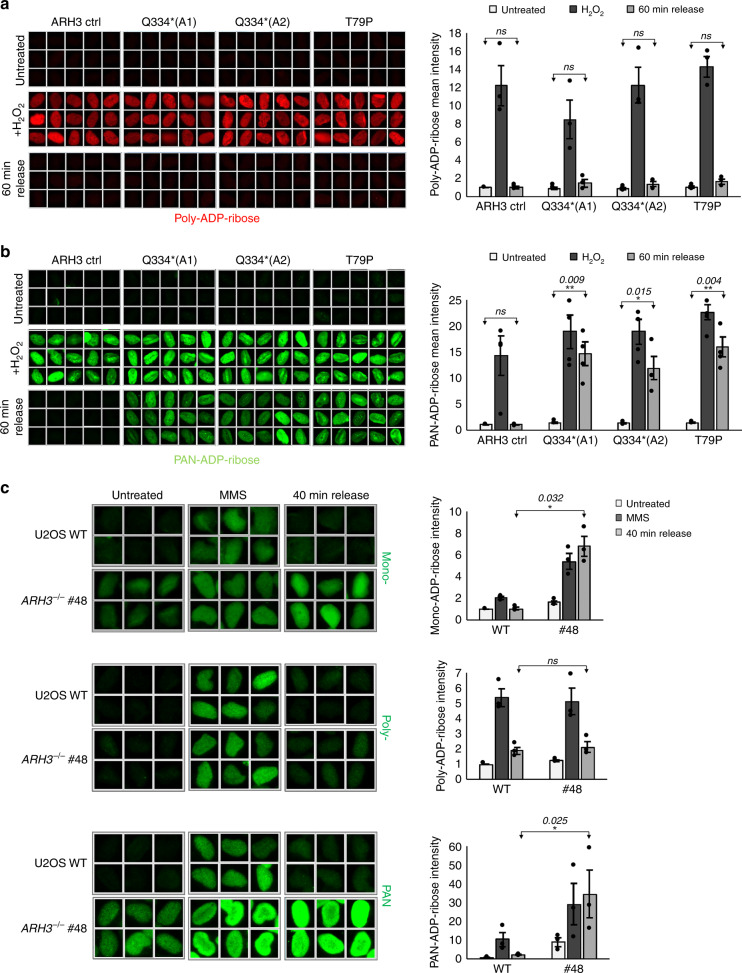
ARH3-defective cells accumulate mono(ADP-ribose) in chromatin during the repair of exogenous DNA damage. Levels of ADP-ribosylation were measured by indirect immunofluorescence in control and patient fibroblasts before, immediately after 10 min treatment with 150 μM H_2_O_2_ on ice, and after a 60 min repair period in H_2_O_2_-free medium. Cells were pre-extracted with detergent prior to fixation and staining with an anti-poly-ADP-ribose antibody (**a**) or an anti-PAN-ADP-ribose binding reagent that detects both mono- and poly-ADP-ribose (**b**). **c** Levels of ADP-ribosylation were analysed by indirect immunofluorescence in wild-type and *ARH3*^*−/−*^ U2OS cells before, immediately after 20 min treatment with 0.2 mg/ml MMS, and after a 40 min repair period in MMS-free medium. Cells were pre-extracted with detergent prior to staining with anti-mono-ADP-ribose binding reagent, anti-poly-ADP-ribose antibody, or anti-PAN-ADP-ribose binding reagent. Representative ScanR images and quantification using ScanR software for **a**, **b**, and **c** are shown. Data are the mean ± SEM of at least three biologically independent experiments; *n* = 3 for **a**, **c**, and *n* = 4 for **b**. Statistical analysis (two-tailed *t*-test) is indicated (**P < 0.01; *P < 0.05; ns not significant).

The lack of a SSBR defect in ARH3-defective cells was surprising because ARH3 has been reported to promote poly(ADP-ribose) catabolism; a process known to be important for SSBR^20,21,25^. We therefore measured directly the level of poly(ADP-ribose) in nuclear chromatin in control and ARH3 patient cells before and after SSB-induced oxidative stress, using an antibody directed against the polymer. The H_2_O_2_-induced signal detected by poly(ADP-ribose)-specific antibody declined to background levels in both control and ARH3 patient cells (Fig. 2a). These data suggest that the catabolism of poly(ADP-ribose) in chromatin is largely unaffected in the *ARH3*-mutated patient cells, likely explaining why both XRCC1 recruitment and SSBR rates are largely normal in these cells.

In contrast to the results obtained with anti-poly(ADP-ribose) antibody we detected striking differences in the level of ADP-ribose signal using an anti-PAN-ADP-ribose reagent that detects terminal ADP-ribose moieties and thus both mono- and poly(ADP-ribose)^26^. While the level of ADP-ribose was similar in the chromatin of control fibroblasts and *ARH3*-mutated patient cells immediately after treatment with H_2_O_2_, this level failed to decline in the ARH3 patient cells during a subsequent 60 min incubation in H_2_O_2_-free medium (Fig. 2b). This was not the case in the control cells, in which ADP-ribose levels dropped almost to background during the same time period (Fig. 2b). Similar results were observed in *ARH3*^*−/−*^ U2OS cells, confirming that the persistent ADP-ribose was a result of the loss of ARH3 (Supplementary Fig. 3).

The difference between the anti-poly(ADP-ribose) antibody and the anti-PAN-ADP-ribose reagent in detection of residual ADP-ribose in ARH3-defective cells did not reflect differences in their ability to detect poly(ADP-ribose) chains. This is because both detection reagents detected similar ADP-ribose signals immediately after H_2_O_2_, and both readily detected poly-ADP-ribosylated PARP1 by western blotting (Fig. 2a, b and Supplementary Fig. 4a). Consequently, the above results suggest that it is primarily mono(ADP-ribose) that persists and accumulates in ARH3-defective cells in response to DNA damage. Indeed, consistent with this conclusion, the residual ADP-ribose signal in ARH3 patient cells was detected by an anti-mono(ADP-ribose) detection reagent^26^, the specificity of which was confirmed by western blotting (Supplementary Fig. 4a and 4b). Notably, elevated levels of ADP-ribose were also detected in the chromatin of *ARH3*^*−/−*^ U2OS cells following treatment with MMS, and this ADP-ribose was again detected by mono(ADP-ribose) specific but not poly(ADP-ribose) specific, detection reagent (Fig. 2c).

### Endogenous mono(ADP-ribosylation) in *ARH3*-mutated cells

**Fig. 3:**
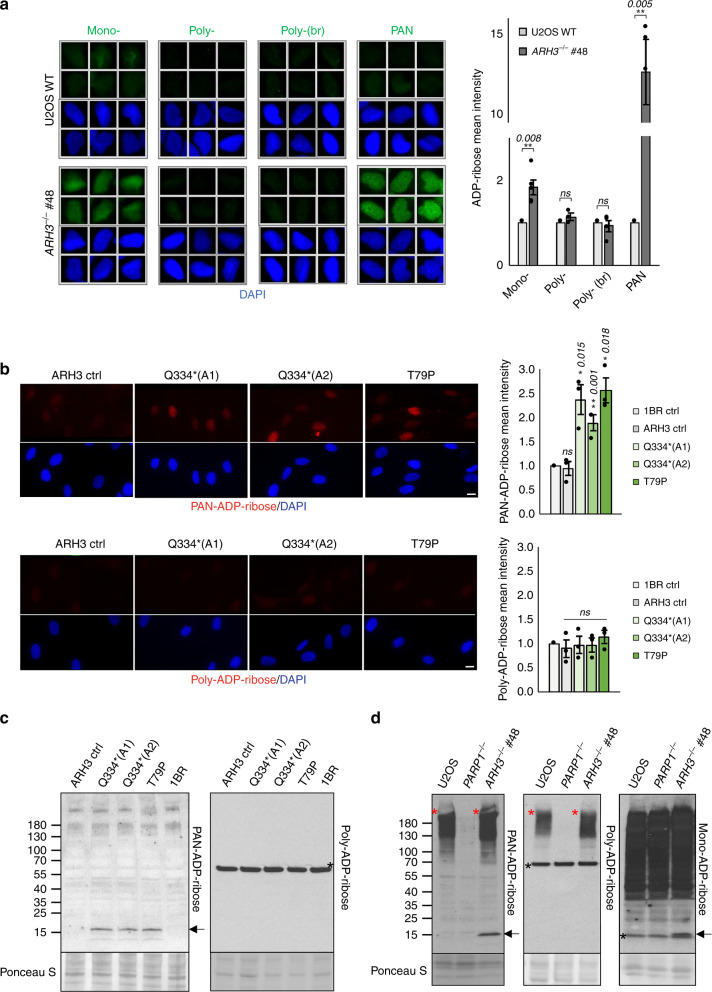
ARH3-defective cells accumulate endogenous mono(ADP-ribosylated) histones. **a** Endogenous levels of ADP-ribosylation were analysed by indirect immunofluorescence in wild-type and *ARH3*^*−/−*^ U2OS cells by detergent pre-extraction prior to fixation and staining with anti-mono-ADP-ribose binding reagent, anti-poly-ADP-ribose antibody, anti-poly-ADP-ribose binding reagent (br), and anti-PAN-ADP-ribose binding reagent. Representative ScanR images (left) and quantification (right) are shown. **b** Endogenous chromatin ADP-ribosylation in control and patient fibroblasts measured after detergent pre-extraction and staining with anti-PAN-ADP-ribose binding reagent or anti-poly-ADP-ribose antibody. Quantification (right) and representative images (left) are shown; scale bar, 10 μM. Quantification for **a** and **b** was done by using ScanR software. Data are the mean ± SEM of *n* = 5 for **a** and *n* = 3 for (**b**) biologically independent experiments. Statistical analysis (two-tailed *t*-test) is indicated (*P < 0.05; **P < 0.01; ns not significant). **c** Endogenous ADP-ribosylation in control, ARH3 patient and XRCC1 patient fibroblasts was detected by western blotting using anti-PAN-ADP-ribose binding reagent or anti-poly-ADP-ribose antibody. The arrow denotes mono(ADP-ribosylated) histones and the black asterisk is a nonspecific band. **d** Endogenous ADP-ribosylation in wild-type, *PARP1*^−*/−*^ and *ARH3*^−/−^ U2OS cells was detected by western blotting using anti-PAN-ADP-ribose binding reagent, anti-poly-ADP-ribose antibody, or anti-mono-ADP-ribose binding reagent. Arrows and the black asterisk are as above, and the red asterisk denotes poly(ADP-ribosylated) proteins (most likely ribosylated PARP1) not bound to chromatin.

We noted in the above experiments that the level of ADP-ribose was elevated in the chromatin of *ARH3*^*−/−*^ U2OS cells even without treatment with exogenous genotoxin (Fig. 2c and Supplementary Fig. 3). This endogenous ADP-ribose was also detected by anti-mono(ADP-ribose) detection reagents but not by two different anti-poly(ADP-ribose) antibodies, further implicating mono(ADP-ribose) as the primary chromatin mark accumulating in *ARH3*^*−/−*^ U2OS cells (Fig. 3a) and consistent with the role of ARH3 as a mono(ADP-ribosylhydrolase)^6,7^. Elevated endogenous ADP-ribose was also observed in ARH3 patient cells, albeit to a lesser extent than in *ARH3*^*−/−*^ U2OS cells (Fig. 3b). This lesser extent of elevated ADP-ribose in the patient cells most likely reflects the small amount of residual ARH3 in these cells, and was below the detection limit of the mono(ADP-ribose) detection reagent. However, that this signal reflected mono(ADP-ribose) was again suggested by its detection by the PAN-ADP-ribose, but not poly(ADP-ribose), reagents (Fig. 3b).

We also detected the elevated endogenous ADP-ribose in the ARH3 patient and *ARH3*^*−/−*^ U2OS cells by western blotting, which based on their electrophoretic mobility implicated histones as the ADP-ribosylated proteins (Fig. 3c, d, arrows). In agreement with our immunofluorescence data, the ADP-ribosylated proteins were detected by anti-PAN-ADP-ribose and anti-mono(ADP-ribose) detection reagents, but not by poly(ADP-ribose)-specific reagents. Histones have been identified previously as cellular targets of ADP-ribosylation, primarily on serine residues that are the substrates of ARH3, both before and after exogenous DNA damage^27–29^. The electrophoretic mobility of the ADP-ribosylated protein/s detected by the PAN-ADP-ribose detection reagent was closest to that of H2B and H3, albeit with a slightly reduced electrophoretic mobility that might reflect the impact of the ADP-ribose (Supplementary Fig. 5a, red asterisks). A slowly migrating polypeptide of reduced mobility was detected in *ARH3*^*−/−*^ U2OS cells even by anti-H2B antibody, suggesting that this ribosylated histone might accumulate in ARH3-defective cells at relatively high levels (Supplementary Fig. 5a, red asterisks). It should be noted that, in U2OS cells, we also detected some high molecular weight ADP-ribosylated proteins that were present independently of ARH3 status (Fig. 3d, red asterisks). Based on their extraction from permeabilised nuclei by detergent these proteins were not core components of chromatin (Supplementary Fig. 5b), and based on their electrophoretic mobility and complete absence from *PARP1*^*−/−*^ U2OS cells were most likely auto-ribosylated PARP1 (Fig. 3d).

Collectively, our results suggest that ARH3 mutation or loss leads to the accumulation of mono(ADP-ribosylated) histones at sites of endogenous SSBR. This mono(ADP-ribose) may be a product of the degradation of poly(ADP-ribose) by PARG, the activity of which leaves a residual protein-proximal ADP-ribose requiring ARH3 activity for removal. Alternatively, or in addition, it may reflect de novo chromatin mono(ADP-ribosylation) at such sites. Consistent with the latter, short incubation (30 min) with PARG inhibitor did not measurably affect the level of endogenous ADP-ribose detected in *ARH3*^*−/−*^ cells by mono(ADP-ribose) specific detection reagent, despite the expected elevation in poly(ADP-ribose) during S phase^30^, although long term suppression of PARG activity is required to confirm this (Supplementary Fig. 5c).

### Chromatin scars induced during cell proliferation

**Fig. 4:**
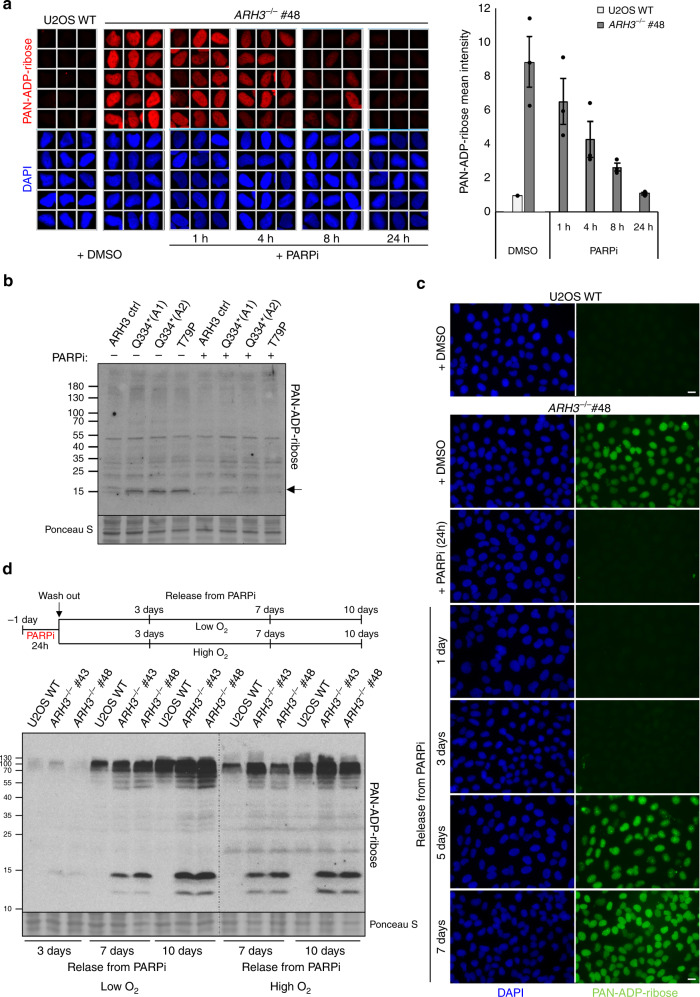
The elevated ADP-ribose in ARH3-defective cells is an erasable chromatin ‘scar’ at the sites of endogenous DNA repair. **a** Endogenous ADP-ribose levels in the chromatin of *ARH3*^*−/−*^ U2OS cells treated with vehicle (DMSO) or 10 μM PARP inhibitor (PARPi) for the indicated times. Representative ScanR images are shown (left) and the mean ADP-ribose intensity relative to untreated wild-type control is quantified (right). Data are the mean ± SEM of three biologically independent experiments. **b** Histone ADP-ribosylation in control and ARH3 patient fibroblasts before and 48 h after treatment with PARPi, measured by western blotting using anti-PAN-ADP-ribose binding reagent. Mono(ADP-ribosylated) histones are indicated by an arrow. **c** *ARH3*^*−/−*^ U2OS cells were treated with DMSO vehicle or 10 μM PARPi for 24 h, washed, and then incubated in PARPi-free medium for the indicated periods. Cells were pre-extracted with detergent prior to fixation and ADP-ribose levels detected with anti-PAN-ADP-ribose binding reagent. Representative images are shown. Scale bar, 20 μM. **d** Wild-type and *ARH3*^*−/−*^ U2OS cells were treated with 10 μM PARPi for 24 h, washed, and then incubated in PARPi-free medium in the presence of low (5%) or high (21%) oxygen for indicated time points. Levels of endogenous ADP-ribosylation were measured by western blotting using anti-PAN-ADP-ribose binding reagent.

The accumulation of ADP-ribose in ARH3-defective cells is an exciting concept because it implicates this modification as a ‘molecular memory’ of endogenous sites of SSBR. Such chromatin scars may thus measure the source, frequency, and location of endogenous SSBs; measurements that have not previously been possible. To explore this idea, we incubated ARH3-defective cells with the PARP inhibitor KU 0058948, which inhibits all three DNA damage stimulated PARP enzymes PARP1, PARP2, and PARP3^31^, for prolonged periods to determine whether the elevated ADP-ribose was lost from chromatin in the absence of ongoing PARP activity. Indeed, the level of ADP-ribose declined to background levels within 24–48 h (Fig. 4a, b), presumably reflecting histone turnover and/or the presence of another, albeit weak, mono(ADP-ribose) hydrolase. Next, we examined whether the mono(ADP-ribose) reappeared in chromatin following removal of the PARP inhibitor, as predicted if this modification is introduced during the repair of endogenous SSBs. Indeed, elevated ADP-ribose levels reappeared in *ARH3*^*−/−*^ cells within 4–5 days of removing PARP inhibitor (Fig. 4c). Next, we exploited this observation to address the source of the endogenous SSBs that trigger the elevated ADP-ribose in chromatin in *ARH3*^*−/−*^ U2OS cells. First, we compared the rate at which the ADP-ribose reappeared in the chromatin of *ARH3*^*−/−*^ cells under conditions of low (5%) or high (21%) oxygen tension, because oxidative stress is a major source of endogenous DNA damage^32^. However, the rate at which histone mono(ADP-ribosylation) was restored in the chromatin of *ARH3*^*−/−*^ cells was unaffected by oxygen levels, suggesting that oxidative DNA lesions were not a major source of ADP-ribose in chromatin over the time course of these experiments (Fig. 4d and Supplementary Fig. 6a).

**Fig. 5:**
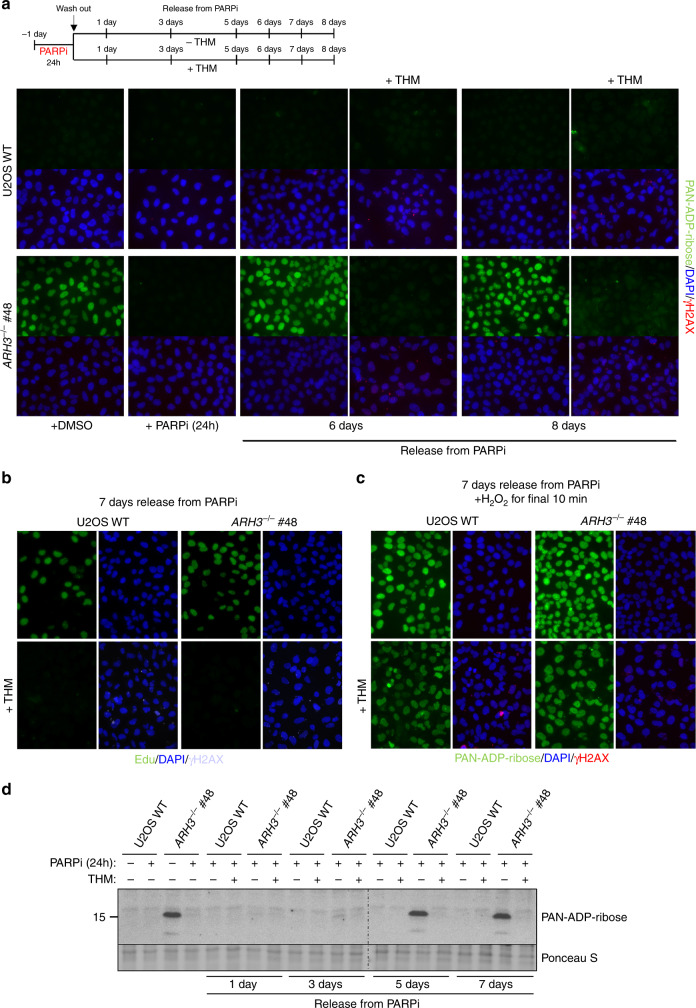
The ADP-ribose chromatin scars in ARH3-defective cells result from S phase DNA damage. **a** Wild-type and *ARH3*^*−/−*^ U2OS cells were treated with DMSO vehicle or 10 μM PARPi for 24 h, washed, and then incubated in PARPi-free medium with or without 2.5 mM thymidine (THM) for the indicated time periods. ADP-ribose levels in chromatin were then measured by indirect immunofluorescence in detergent pre-extracted cells with anti-PAN-ADP-ribose binding reagent. **b** Cells treated as above were pulse-labeled at day 7 after release from PARP inhibitor with 10 μM EdU for the last 20 min prior to harvesting, to confirm that thymidine-induced cell-cycle arrest was successful. **c** Cells treated as in **a** were incubated on day 7 with 150 μM H_2_O_2_ for 10 min and ADP-ribose levels then measured, to confirm that PARP can be activated in thymidine-arrested cells. γH2AX in **a**, **b**, and **c** was used as a marker of replication stress in thymidine treated cells. Representative images for **a**, **b**, and **c** are shown. Scale bar, 20 μM. **d** Wild-type and *ARH3*^−*/−*^ U2OS cells were treated as in **a** and ADP-ribosylated histones detected by western blotting, using anti-PAN-ADP-ribose binding reagent.

Recently, we showed that the primary source of detectable endogenous PARP activity in proliferating cells are unligated Okazaki fragment intermediates of DNA replication^30^. To examine whether these structures might contribute to the endogenous ADP-ribose we compared its reappearance in A*RH3*^*−/−*^ cells that were held at the G1/S boundary by thymidine block with cells that were released into S phase, following removal of PARP inhibitor. Whereas *ARH3*^*−/−*^ cells that were released into S phase restored high levels of ADP-ribose in chromatin within 5–6 days following removal of the PARP inhibitor, the cells that remained in the thymidine block failed to do so (Fig. 5a). This did not reflect an inability to activate PARP in thymidine-arrested cells, because PARP activity was rapidly triggered in these cells by treatment with H_2_O_2_ (Fig. 5b, c). Notably, similar results were observed if ADP-ribose was measured by western blotting, both in *ARH3*^*−/−*^ U2OS cells treated as above and in ARH3 patient fibroblasts in which we regulated entry into S phase by serum starvation (Fig. 5d and Supplementary Fig. 6b). We conclude from these experiments that the major endogenous source of chromatin ADP-ribosylation in proliferating cells are lesions associated with DNA replication, which from our previous work we suggest are unligated Okazaki fragment intermediates of DNA replication^30^. The persistence of ADP-ribose chromatin scars in proliferating neural cells, such as glia, may contribute to the relatively early onset of disease in *ARH3*-mutated patients, during pediatric development^17,18^. However, it is likely that other types of stochastic DNA damage trigger ADP-ribose accumulation over longer time periods in post-mitotic neurones, perhaps explaining the degenerative component of this disease.

### Loss of ARH3 results in deregulated transcription

**Fig. 6:**
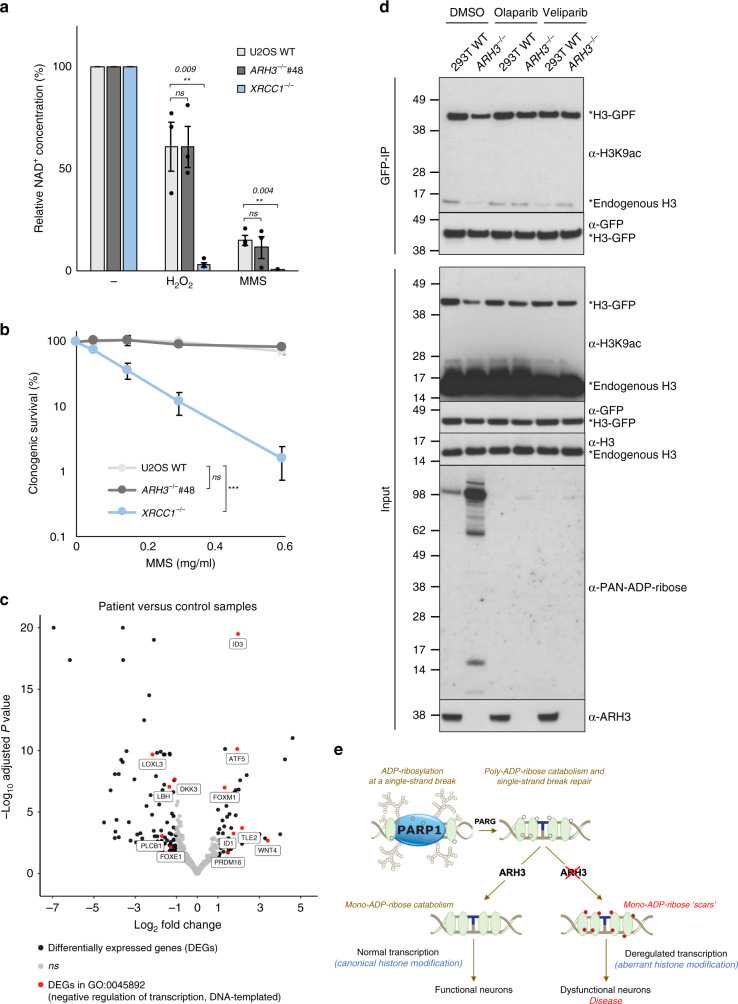
Deregulated transcription in ARH3-defective cells. **a** NAD^+^ depletion in wild-type, *ARH3*^*−/−*^ and *XRCC1*^*−/−*^ U2OS cells following DNA damage. Wild-type, *ARH3*^*−/−*^ and *XRCC1*^*−/−*^ U2OS cells were harvested 45 min after mock-treatment or treatment with either H_2_O_2_ or MMS and NAD^+^ concentrations determined by a chromogenic assay. Data are the mean ± SEM of three independent experiments. Statistical analysis (one-tail *t*-test) is indicated (**P < 0.01; ns, not significant). **b** Clonogenic survival of wild-type, *ARH3*^*−/−*^ and *XRCC1*^*−/−*^ U2OS cells following treatment with the indicated concentrations of MMS. Data are the mean ± SEM of three independent experiments. Statistical analysis (two-way analysis of variance) is shown (ns, not significant; ***P < 0.001; *9.644E-10*). **c** Volcano plot of patient *vs* control transcription profiles showing differentially expressed genes (DEGs; adjusted *P*-value < 0.05, Log_2_ fold change > 1; see Methods). DEGs shown in red are placed in the significantly enriched Gene ontology (GO) category GO:0045892 (negative regulation of transcription, DNA − templated). **d** ADP-ribose chromatin scars result in reduced levels of histone H3K9 acetylation. Wild-type and *ARH3*^*−/−*^ 293 T cells were transfected with plasmid encoding H3-GFP and incubated with DMSO vehicle or with PARP inhibitors (Olaparib or Veliparib), as indicated. 24 h later, GFP-tagged nucleosomes were immunoprecipitated and examined by western blotting for levels of H3K9 acetylation, H3-GFP, endogenous H3, ADP-ribose, and ARH3. Input samples are also shown (bottom panels). **e** A model for the formation and impact of persistent mono-ADP-ribose scars in neurological disease. PARP1 is activated at endogenous SSBs, modifying itself and nearby histones (green ovals) with mono-ADP-ribose (single open circles) and/or poly-ADP-ribose (chains of open circles). Following the completion of SSBR, the de novo mono-ADP-ribose moieties and/or those resulting from PARG activity remain. The mono-ADP-ribose is removed by ARH3 in normal cells, but persists in ARH3 patient cells, forming a chromatin ‘scar’ and providing a ‘memory’ of previously repaired SSBs. The persistent mono-ADP-ribose in ARH3-defective chromatin impedes local histone acetylation and likely other nearby histone modifications, resulting in a perturbed histone code, deregulated transcription, and cellular dysfunction.

Finally, we addressed the mechanism by which the persistent ADP-ribose chromatin scars might trigger cellular dysfunction. It has been reported that ARH3 patient cells undergo NAD^+^ depletion and poly(ADP-ribose)-induced cell death in response to exogenous DNA damage^19^. However, we failed to detect any difference in the level of NAD^+^ depletion or cellular sensitivity of ARH3-defective cells following exogenous DNA damage (Fig. 6a, b, Supplementary Fig. 7a, 7b and 7c). In contrast, we did detect significant differences between ARH3 patient cells and control cells in transcriptome analyses using RNA sequencing, which identified 147 genes that were differentially expressed and of which negative transcriptional regulators comprised a significant GO category (Fig. 6c and Supplementary Fig. 7d). Consequently, we considered the possibility that the ADP-ribose ‘scar’ might disrupt gene transcription, because it has been shown in vitro that the ADP-ribosylation of core histones can prevent other types of chromatin modification, such as the acetylation of H3K9^33^. Indeed, the reverse is also true, in that H3K9 acetylation can prevent the ADP-ribosylation of H3S10, suggesting that these two adjacent histone modifications are mutually exclusive^34^.

To examine this possibility, we transiently-transfected wild-type and *ARH3*^*−/−*^ 293 T cells with GFP-tagged histone H3 and recovered the tagged histone 24 h after transfection, thereby allowing recovery of nascent nucleosomes recently incorporated into chromatin. 293 T cells were employed for these experiments because of their efficiency of transfection, to enable the recovery of sufficient GFP-tagged nascent nucleosomes to detect their histone modifications. These experiments indeed suggested that GFP-tagged nucleosomes in *ARH3*^*−/−*^ cells possessed lower levels of H3-GFP K9 acetylation than did those recovered from wild-type cells (~43% of wild-type levels) (Fig. 6d, top and bottom panels). Reduced K9 acetylation in *ARH3*^*−/−*^ cells was also observed for the endogenous histone H3 that coprecipitated with the GFP-tagged nucleosomes, ruling out that this was an artefact of the overexpressed histone (Fig. 6d, top panel). Importantly, incubation with the PARP inhibitors Olaparib or Veliparib, two highly efficient inhibitors of PARP1, restored levels of K9 acetylation in H3-GFP in *ARH3*^*−/−*^ cells confirming that the reduced acetylation was the result of aberrant ADP-ribosylation (Fig. 6d, top and bottom panels). Collectively, these data suggest that persistent histone ADP-ribosylation results in perturbations in other types of chromatin modification, identifying a possible source of pathogenicity in ARH3-mutated disease. It will now be of interest to determine whether perturbations in histone modification are present in ARH3 patient-derived cells and in vivo, and if so whether such perturbations trigger pathological changes in transcription profile.

In summary, we demonstrate here that ARH3 mutations associated with human neurological disease result in the accumulation of ADP-ribose scars in chromatin that are a molecular memory of endogenous sites of SSBR. Our data suggest that the ADP-ribose chromatin scars prevent normal levels of chromatin acetylation, which we suggest in turn result in deregulated transcription (Fig. 6e).

## Methods

### Antibodies and chemicals

Primary antibodies used in this study were as follows: rabbit polyclonal anti-ARH1 (Invitrogen; PA5-80322), rabbit polyclonal anti-ARH2 (Novus Biologicals; NBP2-39073), rabbit polyclonal anti-ARH3 (Sigma; HPA027104), rabbit polyclonal anti-XRCC1 (Millipore; ABC738), rabbit polyclonal anti-PARP2 (Active Motif; 39743), rabbit polyclonal anti-histone H2A (Abcam; ab13923), rabbit polyclonal anti-histone H2B (Abcam; ab1790), rabbit monoclonal anti-histone H3 (Millipore; 05-928) or rabbit polyclonal anti-histone H3 (Upstate; 07-690), rabbit monoclonal anti-histone H3K9ac (Cell Signaling; #9649), rabbit polyclonal anti-poly-ADP-ribose (Trevigen; 4336), rabbit Fc-fused anti-poly-ADP-ribose binding reagent (Millipore; MABE1031), rabbit Fc-fused anti-PAN-ADP-ribose binding reagent (Millipore; MABE1016), rabbit Fc-fused anti-mono-ADP-ribose binding reagent (Millipore; MABE1076), rabbit polyclonal anti-GFP (Abcam; ab290), mouse monoclonal anti-PARP1 (Santa Cruz; sc-8007), mouse monoclonal anti-PARG (Millipore; MABS61), mouse monoclonal anti-histone H1 (Santa Cruz; sc-393358), mouse monoclonal anti-histone H4 (Cell Signaling; #2935), mouse monoclonal anti-γH2AX (Millipore; 05-636), and rat polyclonal anti-α-tubulin (Abcam; ab6160). Secondary antibodies employed for western blotting were HRP-conjugated goat anti-rabbit (Bio-Rad; 170-6515), goat anti-mouse (Bio-Rad; 170-6516), and rabbit anti-rat (Abcam; ab6734) and for indirect immunofluorescence were goat anti-mouse or goat anti-rabbit Alexa 488 (Invitrogen; A-11001 and A-11008) and donkey anti-mouse or goat anti-rabbit Alexa 568 (Invitrogen; A-10037 and A-11011). PARP inhibitor (PARPi; Axon; KU 0058948), Olaparib (Cayman Chemical; AZD 2281), Veliparib (Selleckchem; ABT-888), and PARG inhibitor (PARGi; Sigma; PDD00017273) were dissolved in dimethyl sulfoxide (DMSO) (Sigma–Aldrich) to a working concentration of 10 mM. Hydrogen peroxide (H_2_O_2_), Methyl methansulfonate (MMS), camptothecin (CPT) and thymidine (THM) were obtained from Sigma–Aldrich (H1009, 129925, C9911, and T1895). H_2_O_2_ was dissolved directly into serum-free medium, MMS was dissolved into culture medium, 10 mM CPT solution was prepared in DMSO and 200 mM THM in water. Final concentrations were as follows*:* 10 μM PARPi, 10 μM PARGi, 0.1 mg/ml and 0.2 mg/ml MMS, 10 μM CPT and 2.5 mM THM or they are indicated in the text.

### Cell lines and culture

The patient-derived primary human fibroblasts A1 and A2 were derived from the affected siblings II–IV–6 & II–IV–7 and harbour the ARH3 homozygous nonsense mutation Q334*, and T79P was from an unrelated affected individual harbouring the ARH3 homozygous missense mutation T79P (Individual II–1, family 3)^17^. The control primary fibroblast cell line ARH3 ctrl was derived from the unaffected father (III-II) of A1 and A2^17^. The unrelated control primary fibroblast 1BR and the XRCC1-mutated patient primary fibroblasts have been described previously^15^. All primary human fibroblasts were cultured in Minimum Essential Media (MEM; Gibco) supplemented with 15% Fetal Bovine Serum (FBS, Gibco; 10270-106), 2 mM L-glutamine (Gibco; 25030-024), and the antibiotics penicillin (100 units/ml) and streptomycin (100 μg/ml) (Gibco; 15140-122) at 37 °C, 5% O_2_ and 5% CO_2._ Human *ARH3*^*−/−*^ (clones #43 and #48), *PARP1*^*−/−*^ (clone #4), and *XRCC1*^*−/−*^ (clone #2) U2OS cell lines have been described previously^7,11,35^. Cells were grown in Dulbecco’s Modified Eagle Medium (DMEM, Sigma; D6429) containing 10% FBS and the antibiotics penicillin/streptomycin at 37 °C, 5% O_2_ and 5% CO_2_.

### Generation of 293 T *ARH3*^*−/−*^ cell line

Human embryonic kidney 293 T (ATCC CRL-3216) cells were acquired from ATCC and were grown in DMEM (Sigma; D6429) containing 10% FBS and the antibiotics penicillin/streptomycin at 37 °C and 5% CO_2_. The protocol for generating gene-edited 293 T *ARH3*^*−/−*^ cell line has been used previously for U2OS cells^7^. Briefly, sgRNA 210 (GCGCTGCTCGGGGACTGCGT) and sgRNA 212 (GGGCGAGACGTCTATAAGGC) were cloned into epX459(1.1), pX459 plasmid containing enhanced Cas9 (eSpCas9) v1.1. 293 T cells were transfected with control sgRNA or cotransfected with sgRNAs 210 and 212 (1:1 ratio) using TransIT-LT1 Transfection Reagent (Mirus), following the manufacturer’s instructions. Twenty-four hour after transfection, the cells were selected with 2 μg/mL Puromycin (InvivoGen) for 36 h and seeded on 96-well plates at low densities (0.4 cells/well). Single colonies were propagated, and individual clones were screened by western blotting.

### SDS-PAGE and western blotting

Sub-confluent cell monolayers were washed with PBS and scraped into SDS sample buffer (2% SDS, 10% glycerol, 50 mM Tris-Cl, pH 6.8), denatured for 10 min at 95 °C, and sonicated for 2 × 30 sec using Bioruptor^®^ Pico (Diagenode). Protein concentrations were determined using the BCA assay (Pierce). DTT and bromophenol blue were added to samples, which were subjected to SDS-PAGE (14% or gradient), proteins transferred onto nitrocellulose membrane and detected by relevant specific antibodies combined with horseradish peroxidase-conjugated secondary antibodies. Peroxidase activity was detected by ECL reagent (GE Healthcare) and Medical X-ray Film Blue (Agfa HealthCare).

### siRNA and transfection

Nontargeting siRNA (ON-TARGETplus) and SMARTpool siRNA against ARH3 (Dharmacon) were reverse-transfected into the cells using Lipofectamine RNAiMAX (Invitrogen) according to the manufacturer’s instructions. Cells were harvested 72 h post-transfection, lysed in SDS buffer, and analysed by western Blotting.

### Transfection of histone H3-GPF

293 T WT or *ARH3*^*−/−*^ cells were plated with 0.5 μM Olaparib or 5 μM Veliparib, cultured for three days and transfected in the presence of the drug using Polyfect (Qiagen) with a plasmid expressing H3-GFP for 24 h. The cells were washed with PBS and lysed with Triton X-100 lysis buffer (50 mM Tris-HCl pH 8.0, 100 mM NaCl, 1% Triton X-100) supplemented with 5 mM MgCl_2_, protease and phosphatase inhibitors (Roche), 1 μM PARG inhibitor and 1 μM Olaparib. The lysates were incubated with Benzonase (Sigma) for 20 min at 4 °C, centrifuged at 20,000 × g for 15 min, and the supernatants were collected. Protein concentrations were normalized, then the cell lysates were incubated with GFP-Trap MA magnetic agarose beads (ChromoTek) for 2 h while rotating at 4 °C. The beads were washed several times with Triton X-100 lysis buffer and eluted with 2x NuPAGE LDS sample buffer (Invitrogen) with TCEP (Sigma). The samples were then analysed by Western Blotting.

### Immunofluorescence and microscopy

Cells cultured on glass coverslips were washed with PBS and fixed with 4% formaldehyde (VWR Chemicals; 9713.1000) 10 min at room temperature (RT). Where required (mainly for XRCC1 chromatin retention and chromatin bound ADP-ribose), before fixation, cells were pre-extracted for 2 min on ice in 0.2% Triton X-100. After fixation, cells were washed twice with PBS, permeabilised by a 5 min incubation in ice-cold methanol/acetone solution (1:1), washed and blocked at least 30 min in 10% FBS in PBS. Incubation with the primary antibody (60 min, RT) was followed by washing (3 × 5 min in PBS) and incubation with appropriate fluorescently-labelled secondary antibody (60 min, RT). Coverslips were washed (3 × 5 min in PBS), stained with DAPI (1 μg/ml in water, 2 min), and mounted using VECTASHIELD (Vector Laboratories). EdU labeling was performed using a Click-iT™ EdU Alexa Fluor™ 488 Imaging Kit (Invitrogen) according to the manufacturer’s instructions. Representative pictures of fixed samples were acquired on a Leica DM6000 or DMi6000 fluorescence microscope. Automated wide-field microscopy was performed on an Olympus ScanR high-content screening station equipped with a motorized stage and ×40/0.95NA (UPLSAPO 2 40×) dry objective. Nuclei were identified based on the DAPI signal and total nuclear XRCC1 and ADP-ribose fluorescence intensity was quantified in the region colocalizing with DAPI using ScanR Analysis Software. At least 500 nuclei were counted per condition in three or more independent experiments. Data are represented as mean ± SEM.

### Alkaline comet assay

Alkaline comet assays were performed essentially as described^36^. In brief, resuspended cells were either mock-treated or treated with 100 μM H_2_O_2_ (U2OS cells) or 50 μM H_2_O_2_ (primary human fibroblasts) for 10 min on ice or with the indicated concentration of MMS for 20 min or CPT for 45 min at 37 °C. H_2_O_2_-treated cells were then rinsed in ice-cold PBS and incubated in fresh drug-free media for the desired repair period at 37 °C. Collected cells were then analysed by alkaline comet assay using Comet Assay IV software (Perceptive Instruments). Data are plotted as comet tail moments of 300 cells from three independent experiments (3 experiments × 100 cells).

### Clonogenic survival assay

Clonogenic survival was determined by colony formation assays. Wild-type human U2OS cells and gene-edited derivatives were plated in 10 mm dishes. After 4 h incubation, cells were treated with indicated concentrations of MMS for 15 min at 37 °C, washed twice with PBS, and incubated for 10–14 days. Cells treated with indicated concentration of CPT were incubated with drug-containing media until fixation. 1BR control and patient-derived human fibroblasts were seeded on irradiated feeder cells in 10 mm dishes, treated with MMS 4 h later and incubated for 21 days. Cells were fixed in 100% methanol and stained with crystal violet solution (0.05% crystal violet, 4% formaldehyde, 1% methanol). The surviving fraction at each dose was calculated by dividing the average number of colonies in treated dishes by the average number in untreated dishes.

### In vitro ADP-ribosylation assay

0.5 μg of recombinant Strep-tagged PARP1 or His-tagged PARP3 was mock-ribosylated in the absence of NAD^+^ or auto-ribosylated in the presence of the 0.1 mM NAD^+^ (NEB)(for PARP1) or 200 mM NAD^+^ (for PARP3) in 50 mM Tris-HCl pH 8, 0.8 mM MgCl_2_, 1% glycerol, 1.5 mM DTT, and 40 nM single-stranded oligodeoxyribonucleotide (Eurogentec: 5′-CATATGCCGGAGATCCGCCTCC-3’), in a final volume of 50 μl at room temperature for 30 min. Reactions were then snap frozen in liquid nitrogen and stored at −80 °C until required. Strep-tagged PARP1 was prepared from Sf9 cells using a 1 ml StrepTrap column (GE Healthcare) and His-PARP3 was prepared as described previously^37^.

### Measurement of NAD^+^ levels

NAD^+^ levels in cells were determined by a chromogenic assay as described before^38^. Briefly, 2 × 10^5^ wild-type human U2OS cells or gene-edited derivatives or 2 × 10^5^ wild-type 1BR or patient-derived human fibroblasts were treated with 150 μM H_2_O_2_ or 0.2 mg/ml MMS for 45 min at 37 °C, trypsinized, washed twice with PBS, and lysed in 20 mM sodium bicarbonate, 100 mM sodium carbonate, 0.05% Triton X-100, 10 mM nicotinamide, pH 10.3 by two freeze/thaw cycles. Lysates were added to a 10,000 MWCO centrifugal filter (UFC501024, Merck™) and centrifuged at 14,000 × *g* at 4 °C for 30 min. Half of each lysate was then incubated at 60 °C for 30 min to decompose NAD^+^. Samples were incubated in cycling buffer [100 mM tricine-NaOH (pH 8), 4 mM EDTA, 40 mM NaCl, 1.66 mM phenazine ethosulfate (PES), 0.42 mM thiazolyl blue tetrazolium bromide (MTT), 10% ethanol] at 37 °C for 5 min, and 10 U/ml alcohol dehydrogenase, reconstituted in 100 mM tricine-NaOH (pH 8), was added to drive a cycling reaction at 37 °C for 40 min. The reaction was terminated by addition of NaCl (2 M final concentration) and samples were centrifuged at 14,000 × *g* at 4 °C for 5 min. Reduced MTT was resuspended in 100% ethanol and the absorbance was measured at 570 nm. NAD^+^ concentrations were calculated by subtracting NADH concentrations from concentrations of samples in which NAD^+^ was not decomposed prior to the cycling reaction.

### Transcriptomics

Total RNA was isolated from 5 × 10^5^ control (1BR and ARH3 ctrl) and patient (A1 and A2) fibroblasts by RNeasy Micro Kit (Qiagen) including DNase I treatment, according to the manufacturer’s instructions. The quantity of isolated RNA was measured spectrophotometrically using NanoDrop ND-1000 (NanoDrop Technologies) and its quality was analyzed by Agilent 2100 Bioanalyser (Agilent Technologies). RNA integrity number, which is regarded as criteria for high-quality total RNA, ranged between 8.9 and 9.8. Poly(A) RNA Selection Kit (Lexogen) was used for mRNA extraction followed by library preparation with SENSE Total RNA-Seq Library Prep Kit (Lexogen). Libraries were sequenced on the Illumina NextSeq^®^ 500 instrument using 84 bp single-end configuration. Read quality was assessed by FastQC (http://www.bioinformatics.babraham.ac.uk/projects/fastqc). For subsequent read processing, a bioinformatic pipeline nf-core/rnaseq version 1.3 was used (Phil Ewels. nf-core/rnaseq: nf-core/rnaseq version 1.3. (2019). 10.5281/zenodo.2610144). Individual steps included removing sequencing adaptors with Trim Galore! (http://www.bioinformatics.babraham.ac.uk/projects/trim_galore/), mapping to reference genome GRCh38^39^ (Ensembl assembly version 95) with HISAT2^40^ and quantifying expression on gene level with featureCounts^41^. Per gene mapped counts served as input for differential expression analysis using DESeq2 R Bioconductor package^42^. Prior to the analysis, genes not expressed in at least two samples were discarded. Shrunken log_2_-fold changes using the adaptive shrinkage estimator^43^ were used for differential expression analysis. We supplied experimental model assuming sex of sampled individuals as explanatory variable and condition (patient vs. control) as the main effect with no interaction term between. Genes exhibiting minimal absolute log_2_-fold change value of 1 and statistical significance (adjusted *P*-value < 0.05) between compared groups of samples were considered as differentially expressed for subsequent interpretation and visualization. *P*-values were calculated using Wald test for the GLM coefficients (log2 Fold change) and adjusted with Benjamini and Hochberg (FDR) method. Gene ontology (GO) enrichment analysis was done using gene length bias aware algorithm implemented in goseq R Bioconductor package^44^.

### Statistics, data analysis, and reproducibility

The number of experimental repeats and statistical tests are indicated in the relevant figure legends. All experiments were conducted three times unless stated otherwise. The mean values ± SEM from multiple biologically independent experiments are indicated as appropriate. Statistical analysis for the comparison between two groups was conducted using Student’s *t*-test, while comparisons between multiple groups was conducted using two-way analysis of variance (ANOVA) test. Collection and analysis of microscopic data was automated and free of user bias. Data from high-content ScanR microscope are presented as mean values of mean intensity of at least 500 nuclei per condition ± SEM of three or more biologically independent experiments. Typical pictures of nuclei per condition for each experiment are shown in galleries. The magnification of each random nucleus in a gallery is adjusted and generated by the ScanR software. The molecular markers for all presented western blots are show in kDa and full scans are available in Source data files. Alkaline comet assays were scored blind by one experimenter and subsequently decoded by a second. Data are plotted as comet tail moments of 300 cells from three biologically independent experiments (3 experiments × 100 cells).

### Reporting summary

Further information on research design is available in the Nature Research Reporting Summary linked to this article.

## Supplementary information

Supplementary Information

Reporting Summary

## Data Availability

The RNA-seq datasets generated during the current study have been deposited in ArrayExpress with the Accession number E-MTAB-8391 and could be accessed under this link: https://www.ebi.ac.uk/arrayexpress/experiments/E-MTAB-8391. All the other data supporting the findings of this study are available within the article, its Supplementary information or Source data files and from the corresponding authors upon reasonable request. A reporting summary for this article is available as a Supplementary Information file. Source data are provided with this paper.

## Source data

Source Data 1
Source Data 2
