# Supplementary material for: Pathogenic ARH3 mutations result in ADP-ribose chromatin scars during DNA strand break repair
Source: Nat Commun. 2020 Jul 7;11:3391. doi: 10.1038/s41467-020-17069-9 (PMC7341855; doi:10.1038/s41467-020-17069-9)
Supplement: Supplementary file 1 — Supplementary Information [file 41467_2020_17069_MOESM1_ESM.pdf]

Pathogenic *ARH3* Mutations Result in ADP-ribose Chromatin Scars during DNA Strand Break Repair

Hanzlikova et al.

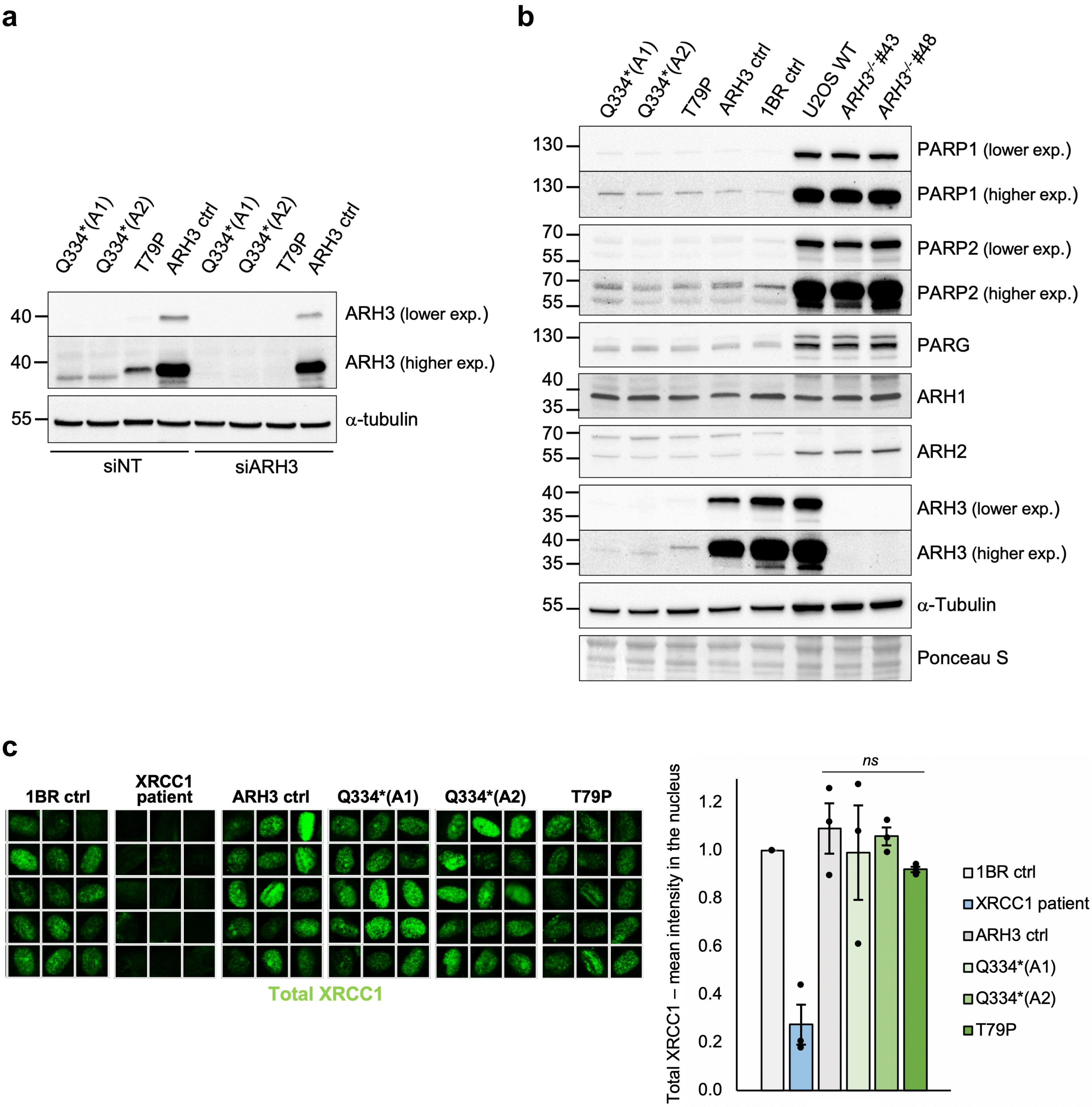

Supplementary Figure 1. Total protein levels in ARH3-defective cells (related to Fig. 1).

a ARH3 protein levels in non-targeting siRNA (siNT) or ARH3 siRNA (siARH3) transfected patient-derived fibroblasts, measured by Western blotting. α-tubulin was employed as a loading control. **b** Total levels of the indicated proteins in control and ARH3 patient fibroblasts and in wild type and *ARH3*$^{-/-}$ U2OS cell lines were analysed by Western blotting. **c,** Total XRCC1 levels were measured by indirect immunofluorescence in formaldehyde fixed control, XRCC1 patient, and ARH3 patient fibroblasts. Representative ScanR images and quantification using ScanR software are shown. Data are the mean +/-SEM of three biologically independent experiments. Statistical analysis (two-tailed *t*-test) is indicated (*ns*, not significant).

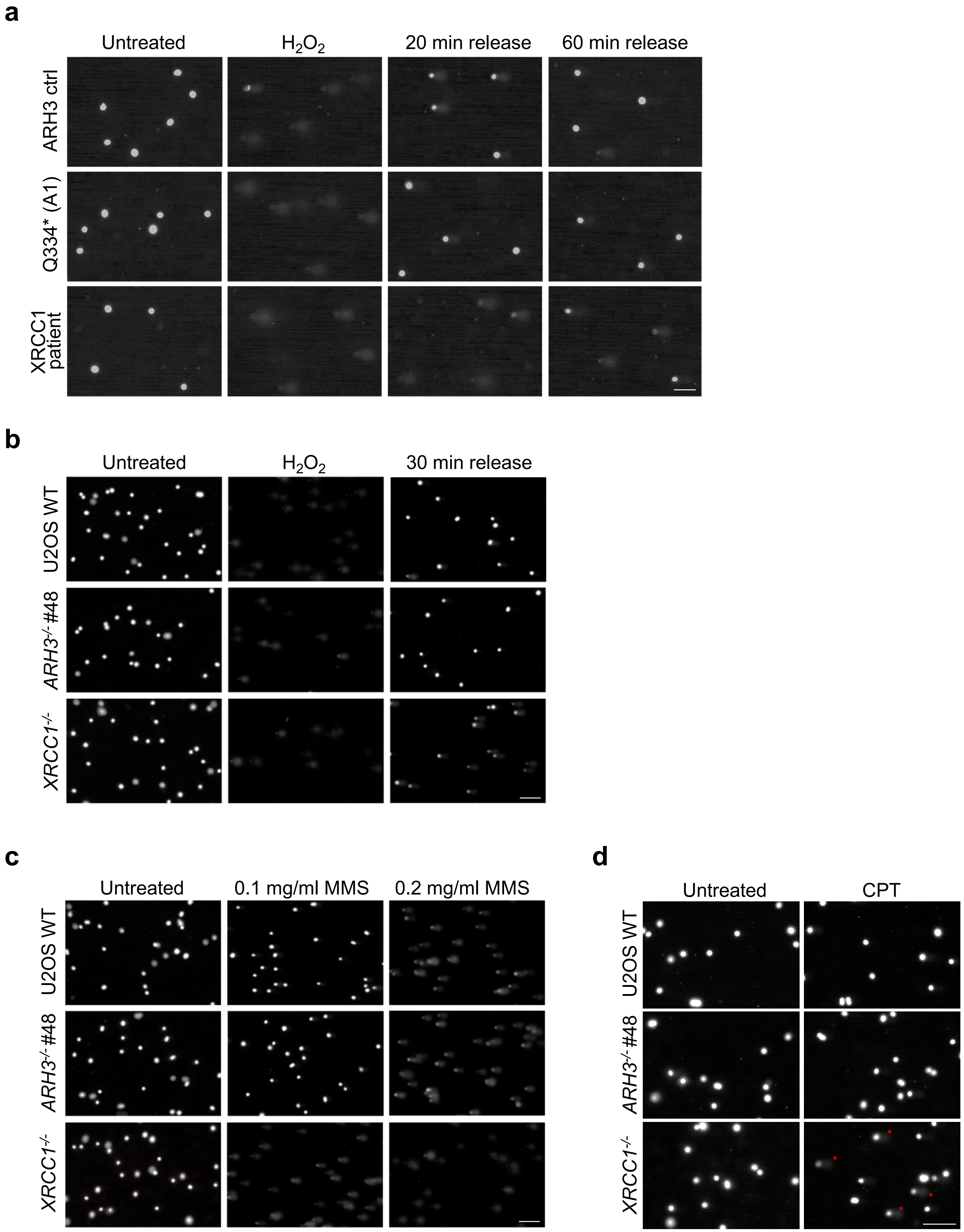

Supplementary Figure 2. Alkaline comet assays in ARH3-defective cells (related to Fig. 1).

Representative pictures of the comet tail moments from the experiments in Figure 1. Alkaline comet assays in the indicated cell lines before, immediately after treatment with 50 μM H_2O_2 on ice, and after the indicated repair periods in H_2O_2-free medium (**a, b**), MMS (**c**) and CPT (**d**). *Red asterisks* highlight examples of cells with visibly increased tail moments (SSBs) in the *XRCC1*$^{-/-}$ defective cells following CPT. Scale bar, 100 μM.

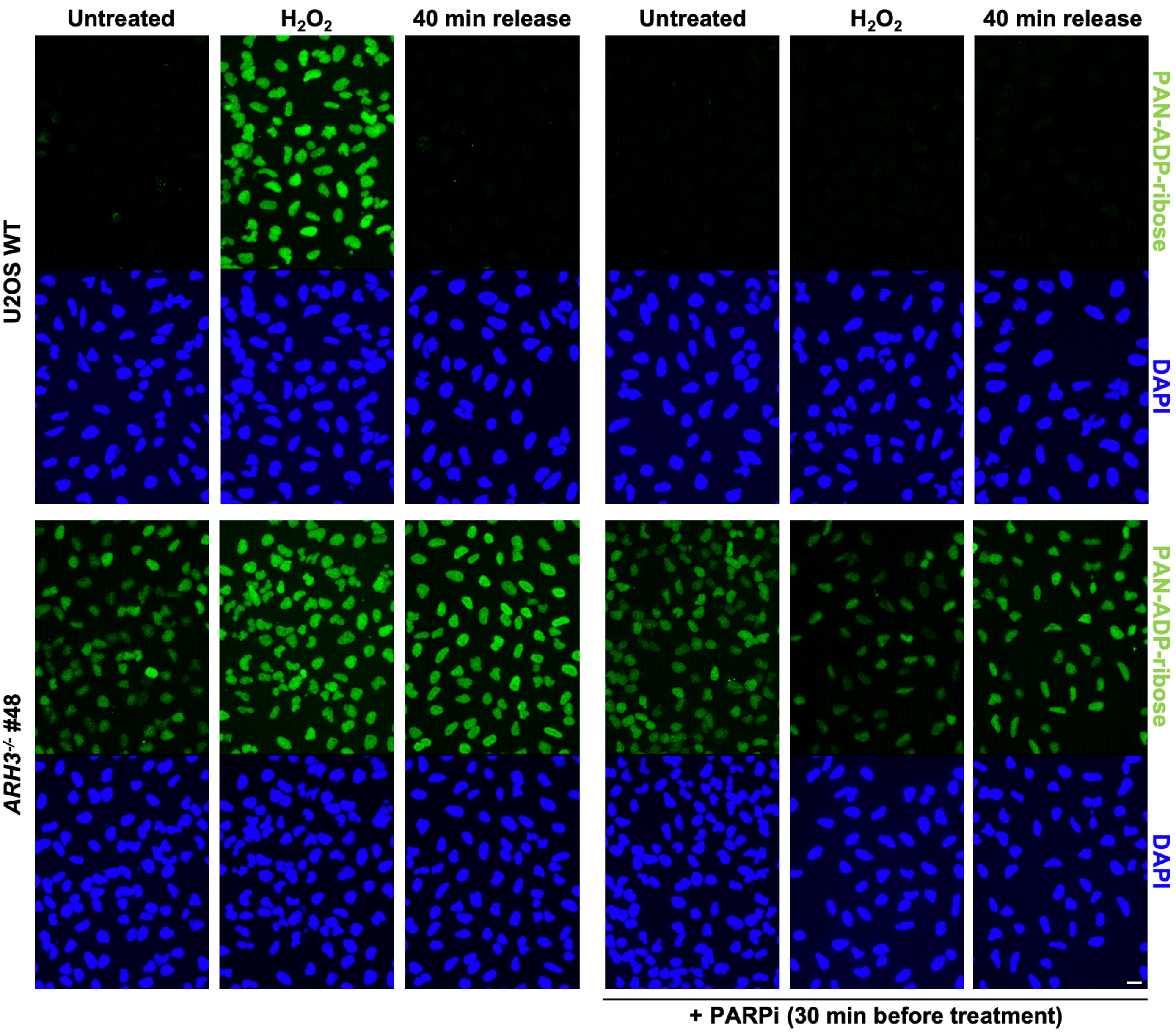

Supplementary Figure 3. ADP-ribose levels in wild type and ARH-3 defective cells following oxidative stress in the presence or absence of PARP inhibitor (related to Fig. 2).

Wild type and *ARH3*$^{-/-}$ U2OS cells were incubated or not for 30 min with 10 μM PARP inhibitor (PARPi), prior to a 10 min incubation with or without 150 μM H_2O_2 on ice. The cells were then either harvested immediately ("H_2O_2") or washed and incubated for a further 40 min in the presence or absence of PARPi, as indicated. Cells were pre-extracted with detergent to remove non-chromatin bound proteins prior to fixation and immunostaining with the anti-PAN-ADP-ribose binding reagent. Representative images are shown. Scale bar, 20 μM.

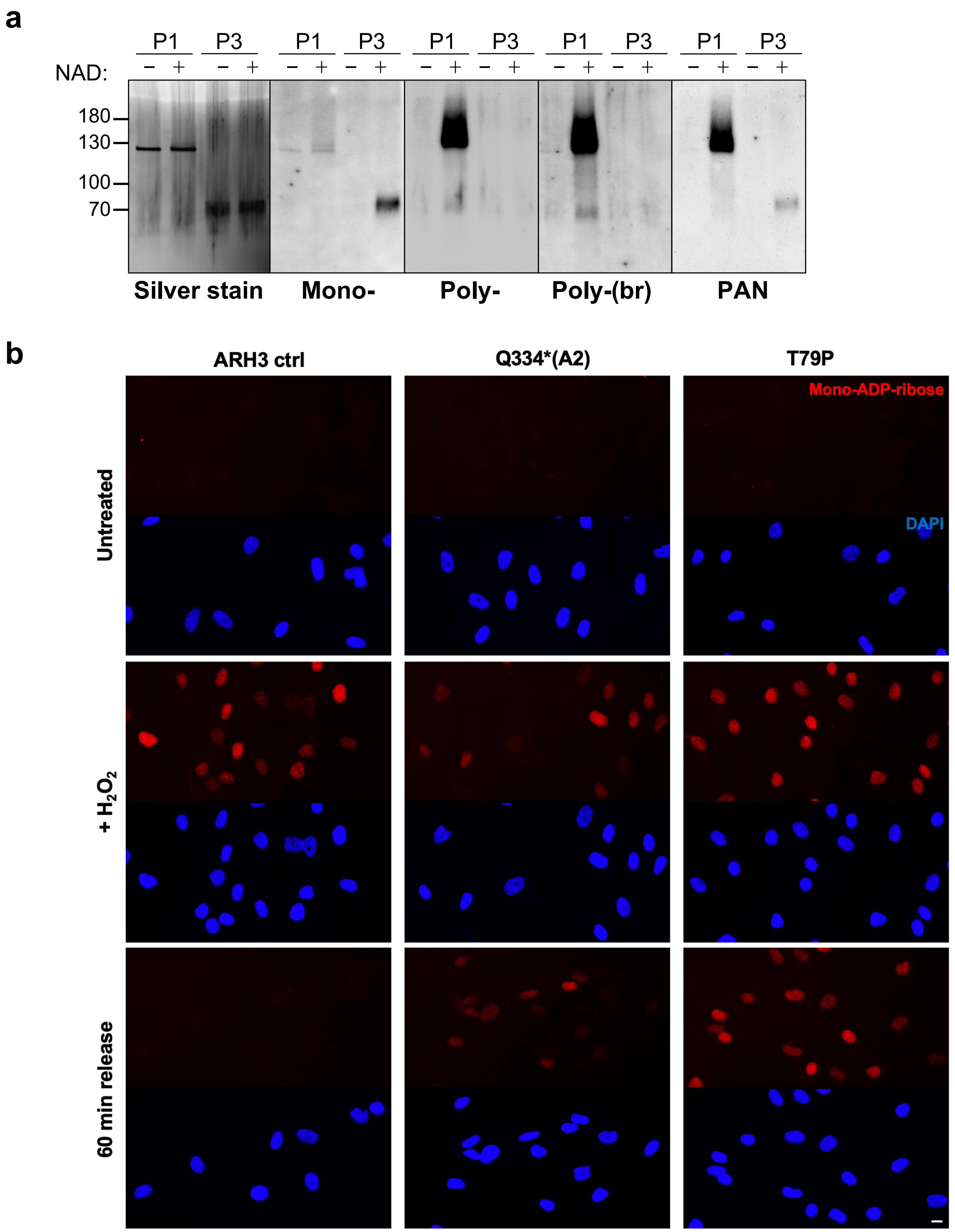

Supplementary Figure 4. Detection of ADP-ribosylation by different reagents (related to Fig. 2).

a Specificity of the ADP-ribose detection reagents employed in this study. 0.5 μg of recombinant PARP1 and PARP3 were auto-*poly*-ADP-ribosylated (PARP1) or auto-*mono*-ADP-ribosylated (PARP3) in the absence ("-") or presence ("+") of either 0.1 μM NAD+ (PARP1) or 0.2 mM NAD+ (PARP3) and fractionated by SDS-PAGE in pentiplicate. One sample was silver stained and four others were incubated with either mono-ADP-ribose binding reagent ("Mono-"), poly-ADP-ribose antibodies ("Poly-"), anti-poly-ADP-ribose binding reagent ("Poly-(br)"), or PAN-ADP-ribose detection reagent ("PAN"). **b** Levels of mono(ADP-ribosylation) were detected by indirect immunofluorescence in control and patient fibroblasts (Q334* and T79P) before ("untreated"), immediately after 10 min treatment with 150 μM H_2O_2 on ice ("+H_2O_2"), and after a 60 min repair period in H_2O_2-free medium. Cells were pre-extracted with detergent prior to fixation and staining with anti-mono-ADP-ribose binding reagent. Representative pictures are shown. Scale bar, 10 μM.

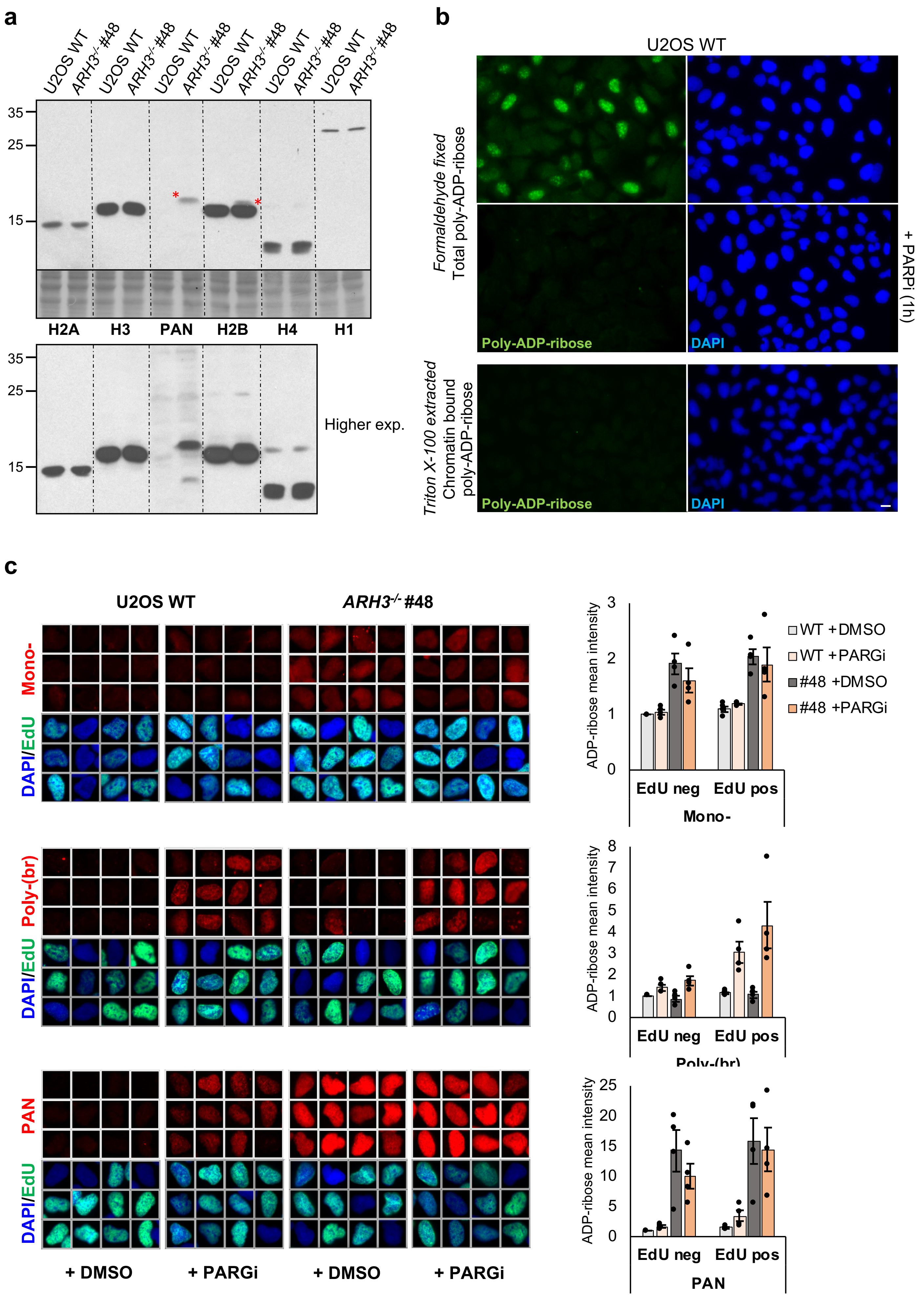

a
U2OS WT
ARH3-/- #48
35
25
15
H2A
H3
PAN
H2B
H4
H1
Higher exp.
b
U2OS WT
Formaldehyde fixed
Total poly-ADP-ribose
Poly-ADP-ribose
DAPI
+ PARPi (1h)
Triton X-100 extracted
Chromatin bound poly-ADP-ribose
c
ARH3-/- #48
Mono-
Poly-(br)
PAN
DAPI/EdU
+ DMSO
+ PARGi
ADP-ribose mean intensity
WT +DMSO
WT +PARGi
#48 +DMSO
#48 +PARGi
EdU neg
EdU pos

Supplementary Figure 5. Mono(ADP-ribosylated) histones accumulate in ARH3-defective cells in the absence of exogenous DNA damage (related to Fig. 3).

a Lysates from wild type and *ARH3*$^{-/-}$ U2OS cells were analysed by Western blotting for ADP-ribosylated proteins using anti-PAN-ADP-ribose binding reagent (“PAN”) and in a parallel with antibodies against the indicated histones. *Red asterisk* indicates most probably mono(ADP-ribosylated) histone H2B. **b** Wild type U2OS cells treated or not for 1 hour with PARP inhibitor (PARPi) were fixed immediately in formaldehyde (to retain all proteins) or were pre-extracted with detergent (Triton X-100) prior to fixation (to retain only chromatin bound proteins), and then stained with anti-poly-ADP-ribose antibody. Representative images are shown. Scale bar, 20 μM. **c** Representative ScanR images (*left*) and quantification (*right*) of ADP-ribose in wild type and *ARH3*$^{-/-}$ U2OS cells incubated for 30 min with 10 μM EdU in the absence or presence of PARG inhibitor (PARGi). Cells were gated according to EdU positivity (“EdU pos”; S phase cells) or EdU negativity (“EdU neg”; G1/G2 cells). Anti-mono-ADP-ribose binding reagent, anti-poly-ADP-ribose binding reagent (br) and anti-PAN-ADP-ribose binding reagent were used. Data are the mean +/-SEM of four biologically independent experiments.

a

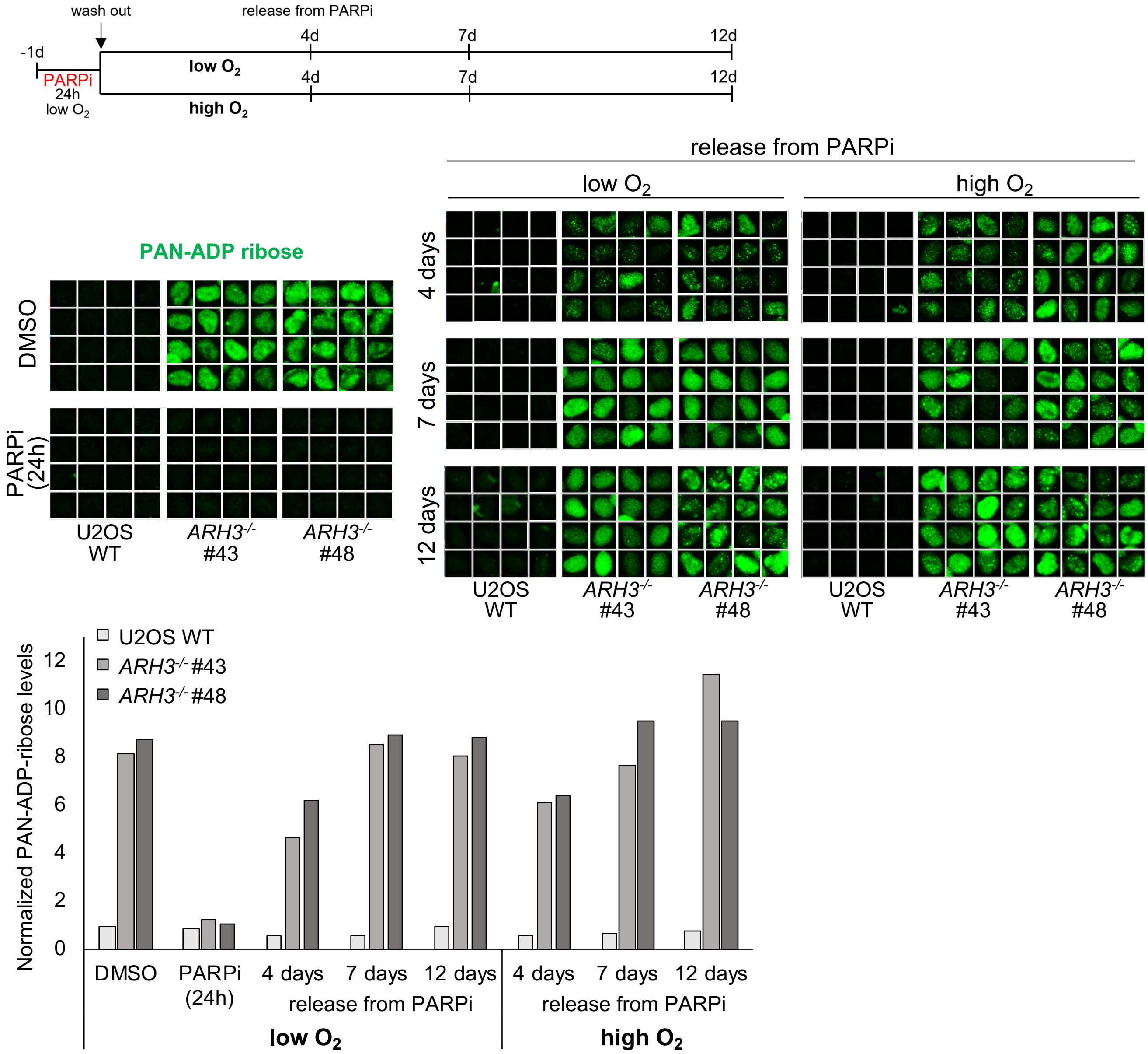

wash out
release from PARPi
-1d
PARPi
24h
low O2
4d
7d
12d
low O2
high O2
PAN-ADP ribose
DMSO
PARPi (24h)
U2OS WT
ARH3-/- #43
ARH3-/- #48
release from PARPi
4 days
7 days
12 days
U2OS WT
ARH3-/- #43
ARH3-/- #48
Normalized PAN-ADP-ribose levels
0
2
4
6
8
10
12
DMSO
PARPi (24h)
4 days
7 days
12 days
release from PARPi
low O2
high O2

b

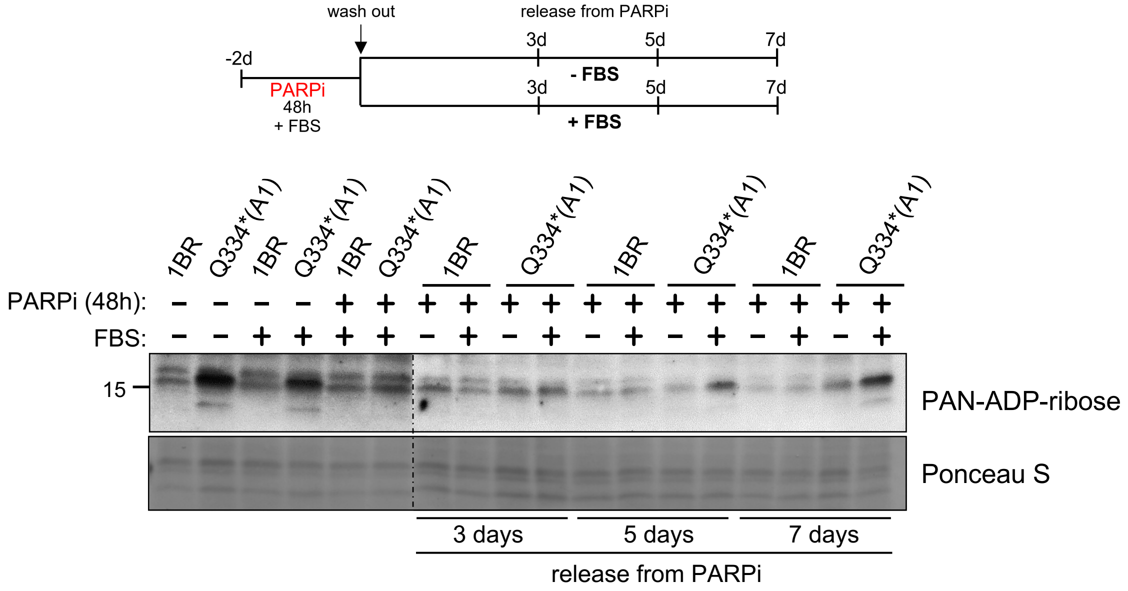

wash out
release from PARPi
-2d
PARPi
48h
+ FBS
3d
5d
7d
- FBS
+ FBS
1BR
Q334*(A1)
PARPi (48h):
FBS:
15
PAN-ADP-ribose
Ponceau S
3 days
5 days
7 days
release from PARPi

Supplementary Figure 6. The elevated ADP-ribose in ARH3-defective cells is an erasable chromatin 'scar' at the sites of endogenous DNA repair (related to Fig. 4 & 5).

a Wild type and *ARH3*$^{-/-}$ U2OS cells were treated with DMSO vehicle or 10 μM PARPi for 24 hours to erase the ADP-ribose chromatin scar (*left panels*) and then washed and released in PARPi-free medium in the presence of low (5%) or high (21%) oxygen for indicated time points (*right panels*). Levels of endogenous chromatin bound ADP-ribosylation were measured by indirect immunofluorescence after detergent pre-extraction prior to fixation and staining with anti-PAN-ADP-ribose binding reagent. Representative pictures and quantification of one experiment using ScanR analysis software are shown. **b** Control and ARH3 patient fibroblasts were treated with DMSO vehicle or 10 μM PARPi for 48 hours, washed, and then released into PARPi-free medium containing 0.75 % serum (-FBS) or 15 % serum (+FBS) to prevent or allow cells to progress into S phase, respectively, for the indicated time periods. ADP-ribosylated histones were detected by Western blotting, using anti-PAN-ADP-ribose binding reagent.

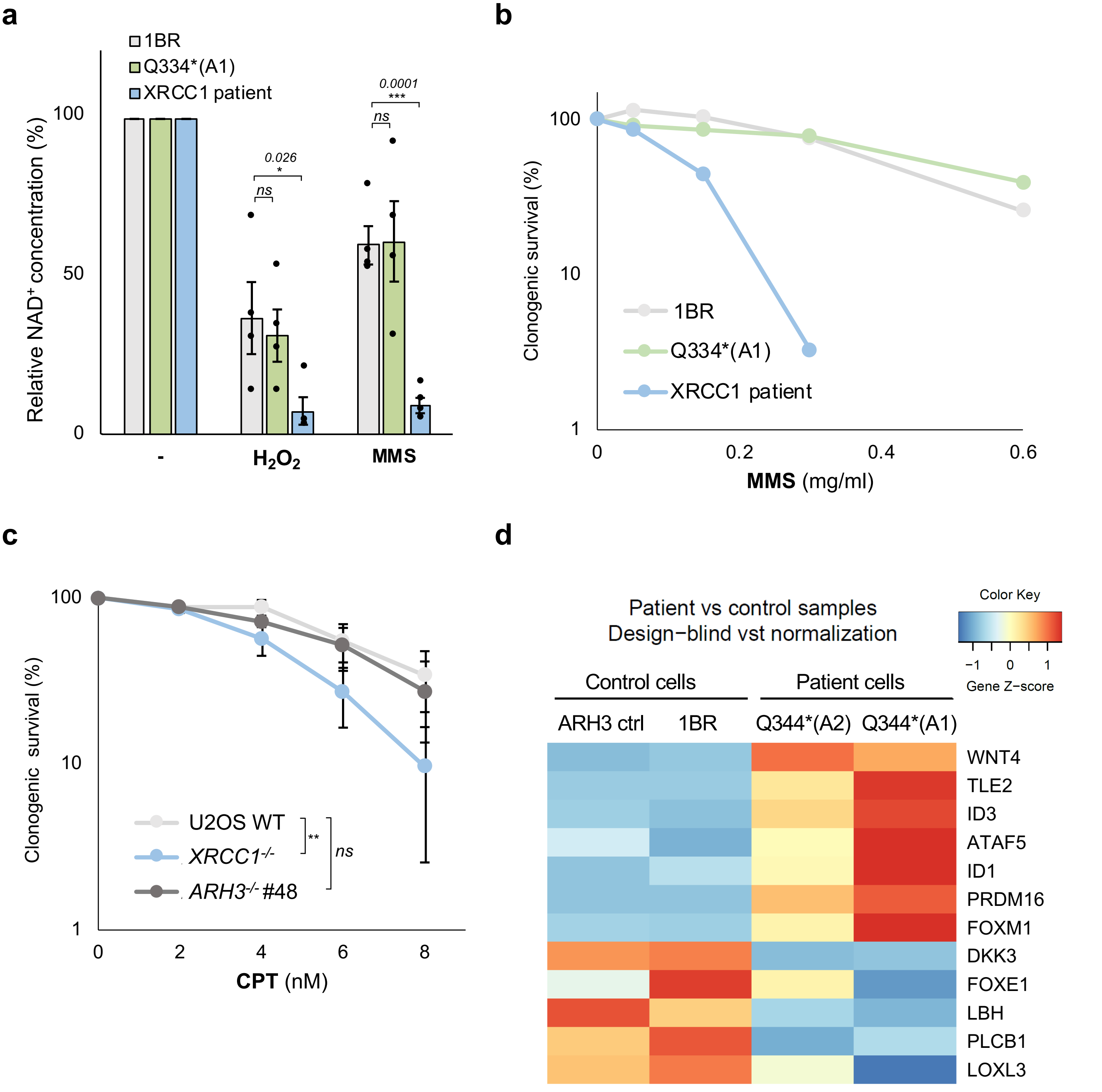

Supplementary Figure 7. Normal sensitivity to genotoxins but altered transcription in ARH3 defective cells (related to Fig. 6).

a NAD^+ depletion in 1BR control, ARH3 patient (A1), and XRCC1 patient fibroblasts following DNA damage. The cells were harvested 45 min after mock-treatment or treatment with either H_2O_2 or MMS and NAD^+ concentrations determined by a chromogenic assay. Data are the mean +/-SEM of four biologically independent experiments. Statistical analysis (one-tail *t*-test) is indicated (*$P < 0.05$; ***$P < 0.001$; *ns*, not significant). **b** Clonogenic sensitivity of 1BR control, ARH3 patient (A1), and XRCC1 patient cell lines to MMS. Data from one experiment. **c** Clonogenic survival of wild-type, *ARH3*$^{-/-}$ and *XRCC1*$^{-/-}$ U2OS cells following treatment with CPT. Data are the mean +/-SEM of three biologically independent experiments. Statistical analysis (two-way analysis of variance) is shown (*ns*, not significant; **$P < 0.01$; *0.006*). **d** Heat map comparison of expression values of genes within the Gene ontology (GO) category GO:0045892 (negative regulation of transcription, DNA−templated) that are differentially expressed in ARH3 patient fibroblasts.
