# Supplementary material for: Pathogenic ARH3 mutations result in ADP-ribose chromatin scars during DNA strand break repair
Source: Nat Commun. 2020 Jul 7;11:3391. doi: 10.1038/s41467-020-17069-9 (PMC7341855; doi:10.1038/s41467-020-17069-9)
Supplement: Supplementary file 2 — Reporting Summary [file 41467_2020_17069_MOESM2_ESM.pdf]

nature research

Corresponding author(s): Hanzlikova and Caldecott

Last updated by author(s): May 6, 2020

Reporting Summary

Nature Research wishes to improve the reproducibility of the work that we publish. This form provides structure for consistency and transparency in reporting. For further information on Nature Research policies, see Authors & Referees and the Editorial Policy Checklist.

Statistics

For all statistical analyses, confirm that the following items are present in the figure legend, table legend, main text, or Methods section.

n/a	Confirmed	
☐	☒	The exact sample size (*n*) for each experimental group/condition, given as a discrete number and unit of measurement
☐	☒	A statement on whether measurements were taken from distinct samples or whether the same sample was measured repeatedly
☐	☒	The statistical test(s) used AND whether they are one- or two-sided<br>*Only common tests should be described solely by name; describe more complex techniques in the Methods section.*
☒	☐	A description of all covariates tested
☒	☐	A description of any assumptions or corrections, such as tests of normality and adjustment for multiple comparisons
☐	☒	A full description of the statistical parameters including central tendency (e.g. means) or other basic estimates (e.g. regression coefficient) AND variation (e.g. standard deviation) or associated estimates of uncertainty (e.g. confidence intervals)
☐	☒	For null hypothesis testing, the test statistic (e.g. *F*, *t*, *r*) with confidence intervals, effect sizes, degrees of freedom and *P* value noted<br>*Give P values as exact values whenever suitable.*
☒	☐	For Bayesian analysis, information on the choice of priors and Markov chain Monte Carlo settings
☒	☐	For hierarchical and complex designs, identification of the appropriate level for tests and full reporting of outcomes
☒	☐	Estimates of effect sizes (e.g. Cohen's *d*, Pearson's *r*), indicating how they were calculated

Our web collection on statistics for biologists contains articles on many of the points above.

Software and code

Policy information about availability of computer code

Data collection	ScanR 3.0.1<br>Comet Assay IV 4.3.1
Data analysis	as above

For manuscripts utilizing custom algorithms or software that are central to the research but not yet described in published literature, software must be made available to editors/reviewers. We strongly encourage code deposition in a community repository (e.g. GitHub). See the Nature Research guidelines for submitting code & software for further information.

Data

Policy information about availability of data

All manuscripts must include a data availability statement. This statement should provide the following information, where applicable:

- Accession codes, unique identifiers, or web links for publicly available datasets
- A list of figures that have associated raw data
- A description of any restrictions on data availability

please see MS for details. All raw data will be made available upon request, and via publicly accessible database (e.g. for RNA-seq)

Field-specific reporting

Please select the one below that is the best fit for your research. If you are not sure, read the appropriate sections before making your selection.

☒ Life sciences ☐ Behavioural & social sciences ☐ Ecological, evolutionary & environmental sciences

For a reference copy of the document with all sections, see nature.com/documents/nr-reporting-summary-flat.pdf

Life sciences study design

All studies must disclose on these points even when the disclosure is negative.

Sample size	Added as requested; please see MS for details, page 17
Data exclusions	no data was excluded
Replication	Added as requested; please see MS for details, page 17.
Randomization	Added as requested; please see MS for details, page 17
Blinding	Added as requested; please see MS for details, page 17

Reporting for specific materials, systems and methods

We require information from authors about some types of materials, experimental systems and methods used in many studies. Here, indicate whether each material, system or method listed is relevant to your study. If you are not sure if a list item applies to your research, read the appropriate section before selecting a response.

Materials & experimental systems

n/a	Involved in the study	
☐	☒	Antibodies
☐	☒	Eukaryotic cell lines
☒	☐	Palaeontology
☒	☐	Animals and other organisms
☒	☐	Human research participants
☒	☐	Clinical data

Methods

n/a	Involved in the study	
☒	☐	ChIP-seq
☒	☐	Flow cytometry
☒	☐	MRI-based neuroimaging

Antibodies

Antibodies used	Antibodies: Primary antibodies used in this study were as follows: rabbit polyclonal anti-ARH1 (Invitrogen; PA5-80322), rabbit polyclonal anti-ARH2 (Novus Biologicals; NBP2-39073), rabbit polyclonal anti-ARH3 (Sigma; HPA027104), rabbit polyclonal anti-XRCC1 (Millipore; ABC738), rabbit polyclonal anti-PARP2 (Active Motif; 39743), rabbit polyclonal anti-histone H2A (Abcam; ab13923), rabbit polyclonal anti-histone H2B (Abcam; ab1790), rabbit monoclonal anti-histone H3 (Millipore; 05-928) or rabbit polyclonal anti-histone H3 (Upstate; 07-690), rabbit monoclonal anti-histone H3K9ac (Cell Signaling; #9649), rabbit polyclonal anti-poly-ADP-ribose (Trevigen; 4336), rabbit Fc-fused anti-poly-ADP-ribose binding reagent (Millipore; MABE1031), rabbit Fc-fused anti-PAN-ADP-ribose binding reagent (Millipore; MABE1016), rabbit Fc-fused anti-mono-ADP-ribose binding reagent (Millipore; MABE1076), rabbit polyclonal anti-GFP (Abcam; ab290), mouse monoclonal anti-PARP1 (Santa Cruz; sc-8007), mouse monoclonal anti-PARG (Millipore; MABS61), mouse monoclonal anti-histone H1 (Santa Cruz; sc-393358), mouse monoclonal anti-histone H4 (Cell Signaling; #2935), mouse monoclonal anti- H2AX (Millipore; 05 636) and rat polyclonal anti- -tubulin (Abcam; ab6160). Secondary antibodies employed for Western blotting were HRP-conjugated goat anti-rabbit (Bio-Rad; 170 6515), goat anti-mouse (Bio-Rad; 170-6516) and rabbit anti-rat (Abcam; ab6734) and for indirect immunofluorescence were goat anti-mouse or goat antirabbit Alexa 488 (Invitrogen; A-11001 and A-11008) and donkey anti-mouse or goat anti-rabbit Alexa 568 (Invitrogen; A-10037 and A-11011). Please note that we have not specific individual dilutions because these must be optmised by each individual user for their own experimental model system.
Validation	all of the antibodies that are important for our conclusions are validated within our own experimental model using appropriate inhibitors (e.g. PAR/MAR) or mutant/deleted cell lines (e.g. ARH3, XRCC1 etc)

Eukaryotic cell lines

Policy information about cell lines

Cell line source(s)	Cell Lines: The patient derived primary human fibroblasts A1 and A2 were derived from the affected siblings II-IV-6 & II-IV-7 and harbour the ARH3 homozygous nonsense mutation Q334*, and T79P was from an unrelated affected individual harbouring the ARH3 homozygous missense mutation T79P (Individual II-1, family 3) ref17. The control primary fibroblast cell line ARH3 ctrl was derived from the unaffected father (III-II) of A1 and A2 ref17. The unrelated control primary fibroblast 1BR and the XRCC1-mutated patient primary fibroblasts have been described previously15. Human ARH3 / (clones #43 and #48), PARP1 / (clone #4) and XRCC1 / (clone #2) U2OS cell lines have been described previously refs 7,11,35.
Authentication	all cell types (U2OS, fibroblasts etc) were authenticated by our central cell culture facility using PCR based fingerprinting as part of their periodic quality and reassurance protocols

Mycoplasma contamination	all cells were confirmed mycoplasma-free by our cell culture facility
Commonly misidentified lines<br>(See ICLAC register)	as indicated above all of the cell lines employed here were confirmed to be free of contaminating cells (e.g. HeLa) by our cell culture facility
